# Further Computations of Quantum Fluid Triplet Structures at Equilibrium in the Diffraction Regime

**DOI:** 10.3390/e28020231

**Published:** 2026-02-16

**Authors:** Luis M. Sesé

**Affiliations:** Independent Researcher, Sucursal 45 Correos, Avda. Valladolid 39, Apartado de Correos 45007, 28008 Madrid, Spain; msese@ccia.uned.es

**Keywords:** quantum fluid triplets, diffraction regime, path integral Monte Carlo, closures, supercritical helium-3, quantum hard spheres

## Abstract

Path integral Monte Carlo simulations and closure computations of quantum fluid triplet structures in the diffraction regime are presented. The principal aim is to shed some more light on the long-standing problem of quantum fluid triplet structures. This topic can be tackled via path integrals in an exact, though computationally demanding, way. The traditional approximate frameworks provided by triplet closures are complementary sources of information that (unexpectedly) may produce, at a much lower cost, useful results. To explore this topic further, the systems selected in this work are helium-3 under supercritical conditions and the quantum hard-sphere fluid on its crystallization line. The fourth-order propagator in the Jang-Jang-Voth’s form (for helium-3) and Cao–Berne’s pair action (for hard spheres) are employed in the corresponding path integral simulations; helium-3 interactions are described with Janzen–Aziz’s pair potential. The closures used are Kirkwood superposition, Jackson–Feenberg convolution, the intermediate AV3, and the symmetrized form of Denton–Ashcroft approximation. The centroid and instantaneous triplet structures, in the real and the Fourier spaces, are investigated by focusing on salient equilateral and isosceles features. To accomplish this goal, additional simulations and closure calculations at the structural pair level are also carried out. The basic theoretical and technical points are described in some detail, the obtained results complete the structural properties reported by this author elsewhere for the abovementioned systems, and a meaningful comparison between the path integral and the closure results is made. In particular, the results illustrate the very slow convergence of the path integral triplet calculations and the behaviors of certain salient Fourier components, such as the double-zero momentum transfers or the equilateral maxima, which may be associated with distinct fluid conditions (e.g., far and near quantum freezing). Closures are shown to yield valuable triplet information over a wide range of conditions, as ascertained from the analyzed centroid structures, which mimic those of fluids at densities higher than the actual ones; thus, closures should remain a part of quantum fluid triplet studies.

## 1. Introduction

The equilibrium description of the quantum monatomic fluid structures at the pair level (n=2), in the diffraction and Bose–Einstein regimes, can be achieved using Feynman’s path integrals (PIs) [1,2] combined with computer simulation methods, i.e., path integral Monte Carlo (PIMC) and path integral molecular dynamics (PIMD) [3,4,5,6,7,8,9,10,11,12,13,14]. (The Fermi–Dirac regime is out of the conventional PI practical applications and requires a special PI formulation [15,16]; see below). The usual PI framework is directly expressed in the coordinate representation and its success at the pair level prompts the interest in undertaking the study of the quantum triplet structural level (n=3) [17,18,19]. Although fluid triplet structures in general cannot be obtained via radiation scattering experiments today [20,21,22,23,24], the equilibrium structures in statistical mechanics behave in a hierarchical manner [25,26,27,28] and the triplet step forward in the quantum domain is needed. This task implies the numerical determination of an involved variety of structural functions, i.e., $g_{3}(r_{12},r_{13},r_{23})$ correlation functions in real space (**r**-space) and $S^{\left(3\right)}(k_{1},k_{2},\phi )$ structure factors in the reciprocal Fourier space (**k**-space) [19,29,30,31,32,33,34,35]. The different nature of these functions depends on the external field Ψ applied that makes them show up.

As a matter of fact, the triplet structural task for monatomic fluids may be regarded as already accomplished in the classical domain [36,37,38,39,40,41,42,43,44,45,46,47,48,49,50,51,52,53,54,55,56,57,58,59], where computer simulation methods (Monte Carlo (MC) and molecular dynamics (MD) [41,45,46,48,51,52,53,54,55,57]) and theories based on integral equations and closures [17,20,25,36,37,38,39,40,42,43,44,45,46,47,49,50,52,53,55,57,58] have been utilized. (Closures are cost-effective theoretical approaches that try, in general, to infer *n*-level structures from the knowledge of the lower-level $n^{\prime }<n$ structures; $n^{\prime }$ and n denote the elemental number of particles involved). Given that PI computer simulations can be regarded as appropriate “translations” of their classical counterparts [4,5,9], the parallel experience accumulated in the classical domain is a precious asset to tackling the quantum triplet structural challenge. The same can be said of closures, which were used to deal with classical and quantum structures alike, regardless of their original motivations and derivations [17,20,25,36,60,61]. Nevertheless, the complexities of the quantum domain have led us to consider some special features that escape the classical analogies [19,32].

Therefore, the quantum fluid triplet program to be followed not only shares the same general reasons that guided the corresponding classical developments, but also must include the new aspects arising from the distinct quantum behaviors. Among the general reasons, one may mention the following: statistical thermodynamics questions beyond the usual pairwise approach, the characterization of the freezing transition, the understanding of the selection between crystal lattice sites, the discussion of glass-forming liquid properties, multiple scattering phenomena, and the calculations of transport coefficients and time-dependent properties (e.g., the dynamic structure factor). Among the new aspects, which in a sense extend the scope of the latter reasons, one may mention the following quantum problems [6,9,14,15,16,17,18,19,62,63,64]: the variety of the distinct fluid responses to external fields, the effect of quantum fluctuations on the formations of crystals and glasses, the role of phonon–phonon interactions in superfluid systems, and the fixing of spin-resolved fluid structures. In connection with these problems, from the scarce initial results on quantum fluid triplets obtained so far, one observes intriguing behaviors of order parameters on the crystallization lines of liquid para-H_2_ [33] and the hard-sphere fluid [34]. In addition, triplet closures have been proven to capture more quantum traits than expected and their usefulness deserves further investigation [19,34,35].

Now, some comments on the difficulties that one encounters when tackling quantum fluid triplets are in order. In the classical and the quantum domains, the dimensionality of the triplet structural functions for a homogeneous and isotropic monatomic fluid is 4-D, with conceptual and computational reasons increasing the complications in the quantum case. The simplest thermal quantum behavior is that of the diffraction effects (i.e., interference phenomena among delocalized atoms, whose magnitude cannot be disregarded); this behavior ignores any possible spin feature of the indistinguishable atoms (or the model one-site particles) composing the fluid. Such diffraction behavior can be observed in every system subjected to low-temperature conditions, but the applied temperatures are to be sufficiently high as to make the spin features negligible [1,2,4,9,12,13,14]. The spin features become fundamental at very low temperatures and add further intricacies to the fluid descriptions: (a) for integer spin atoms, one faces Bose–Einstein exchange statistics (BE) [2,9,10,11,65], and (b) for half-odd-integer spin atoms, one faces Fermi–Dirac exchange statistics (FD) [2,15,16,65]. Typical examples of monatomic systems that can show these exchange regimes are liquid ^4^He (zero-spin atoms, BE) and liquid ^3^He (one-half spin atoms, FD), for which diffraction effects dominate their behaviors so long as the temperatures are T≳2.17 K and T>1 K, respectively [65]. Below these temperature limits, the corresponding BE and FD behaviors cannot be neglected (^3^He even enters the BE regime for T≲2.7 mK) [65].

Interestingly, and focusing on the structural questions, the diffraction and zero-spin BE regimes admit a common general framework in that, by paying attention to their distinct peculiarities, they can be dealt with by using PI in its basic original form [9,10,11,12,13,14,19]. However, nonzero-spin cases require special PI developments; for BE statistics see Reference [66], whereas for FD statistics see the recent works in References [15,16] that use Wigner’s formulation of quantum mechanics [67,68,69] combined with PIMC simulations (WPIMC). (Note that, when studying FD conditions with PI, any proposed method must cope with the so-called “sign problem” [15,16,70,71,72,73]). Contrarily to the pair-level case, the current computational situation for triplet structures is far from being fully affordable [19]. This work concentrates on the thermal quantum diffraction case, for which physically significant triplet calculations can at least be achieved [19,32,33,34,35].

For homogeneous and isotropic monatomic fluids, the thermal quantum diffraction regime may be visualized through the well-known PI image of necklaces composed of beads for representing the actual quantum atoms/particles. There is a one-to-one correspondence between thermally quantum delocalized atoms/particles and necklaces, each of which being composed of a (fixed) number of beads. This allows one to study the quantum fluid and its interactions with external fields Ψ in a very intuitive way [4,9,12,13,14,74,75,76]. By considering different fields, application of linear response theory [14,19,27,28,32,77,78,79] leads to three general classes of quantum fluid structures for the fluid in the absence of Ψ, which are defined in terms of inter-bead/inter-necklace distances in real space: (a) centroid ${\left\{g_{CMn},S_{CM}^{\left(n\right)}\right\}}_{n=2,3,\ldots },$ abbreviated to CMn (a centroid is the “center of mass” of its necklace); (b) instantaneous ${\left\{g_{ETn},S_{ET}^{\left(n\right)}\right\}}_{n=2,3,\ldots },$ abbreviated to ETn; and (c) total thermalized-continuous linear response ${\left\{G_{TLRn},S_{TLR}^{\left(n\right)}\right\}}_{n=2,3,\ldots },$ abbreviated to TLRn. (These three classes can also be identified in the zero-spin BE regime [19,79]).

It is worth stressing that radiation scattering experiments allow one to obtain the pair-level ET2 and TLR2 structures [21,22,23,24]; these structures are defined by the actual atoms composing the sample, because atoms interact effectively with the external field. However, given that the (small) intensity of the triplet contribution is hidden in the whole intensity of the outgoing signal [20], no structural function can be experimentally determined beyond n=2 today. As for the centroid class CMn, although not even the pair CM2 structures can be directly obtained in any experiments (centroids cannot actually couple with any fields), its importance as an intermediate theoretical object cannot be overemphasized, as its usefulness is astonishing (it ranges from the fixing of the fluid equation of state to quantum dynamics studies) [6,7,12,14,19,30,78,79,80,81,82,83,84,85,86,87,88,89,90,91,92,93,94,95].

The foregoing comments on the three distinct quantum structural classes point to the greater complexity of the PI quantum fluid studies as compared to the more reduced scope of their classical counterpart, where only one class of structures ${\left\{g_{Cn},S_{C}^{\left(n\right)}\right\}}_{n=2,3,\ldots },$ abbreviated to Cn, exists. (Note that Cn can be extended further to incorporate, in a formally exact way, the *n*-body direct correlation functions $c_{Cn}$ [37,38,40,45]; this sort of extension may also be added to the quantum classes [14,19,32], although to different degrees of applicability). Nevertheless, these theoretical reasons are just half the issue. The other half includes the practical causes behind the current scarcity of quantum triplet structure applications using “exact” path integral methodologies (in this context, “exact” means “with controllable statistical errors”). Broadly speaking, these causes stem from the extended dimensionality of the PI configuration space (e.g., 3NP, where N stands for the actual number of atoms/particles/necklaces and P is an integer P>1 denoting the number of beads per necklace). This aggravates the computational load when exploring the three quantum classes at the triplet level, for which one notes that (a) any of the structural functions is 4-D; (b) **k**-space calculations require the scanning of a substantial number of sets of wavevectors commensurate with the basic box [45,46]; and (c) apart from the usual scaling with *N* [41,48], the ET3 and TLR3 calculations scale with the number *X* of structurally significant beads (X=P or P/2) [9,12,14,76] as *X* and $X^{3},$ respectively [19,32]. Owing to these difficulties, in the quantum treatments of the classes CM3 and ET3, the introduction of closures and direct correlation functions $c_{3}$ [17,25,38,45,47] may play a significant role [19,29,30,31,32,33,34,35].

The aim of this work, focused on the thermal quantum diffraction regime, is to communicate new results that add more insights into the study of fluid triplet structures. This is a topic that requires large amounts of computational time to obtain statistically significant path integral results. Obviously, the sort of thoroughness usual in the quantum structural studies at the pair level (e.g., see References [96,97]) is currently not possible for triplets and is not attempted herein. Moreover, for the time being, only works by the present author dealing with salient quantum triplet features (equilateral and isosceles), in the real and the Fourier spaces, are available in the literature [19,29,30,31,32,33,34,35]. Therefore, to advance in this topic, this investigation selects two systems that were (partly) analyzed by this author in pilot studies on triplets: helium-3 under supercritical conditions [19,35], and the quantum hard-sphere fluid (QHS) on its crystallization line [19,34]. To understand the present targets pursued, it may be worth mentioning what was achieved in these previous studies.

In Reference [34], quantum hard-sphere fluid triplet questions related to **r**-space were addressed by analyzing the instantaneous and the centroid correlations along the crystallization line (six state points were analyzed). From the path integral results, it was found that the absolute maxima of both classes of equilateral correlations do follow (empirical) decay laws of the general form $g_{3}^{EQ}\left(Max\right)=a\gamma^{-b},$ where *a* and *b* are numerical constants and γ is the degeneracy parameter $\gamma =\lambda_{B}^{3}\rho_{N}$, which is defined in terms of the thermal de Broglie wavelength $\lambda_{B}=h/\sqrt{2\pi mk_{B}T}$ and the number density $\rho_{N}=N/V$ (*m* is the particle mass and V the volume). These are remarkable results that serve as quantum triplet signatures in **r**-space of the QHS crystallization (for conditions γ≲2.7) and suggest that there may exist analogous triplet relationships for real fluids; from this finding, one is also led to expect that triplet structures in **k**-space may contain other types of quantum crystallization signatures [33]. In addition, the role in **r**-space of the intermediate closure AV3, built as the simple average of the Kirkwood superposition [25] and the Jackson–Feenberg convolution approximation [17], was found to be far more important than it might have been anticipated. Thus, as compared to path integral calculations, the intermediate closure AV3 captured a surprising amount of triplet structural traits, for both the instantaneous and the centroid classes; therefore, further research on the limitations of and improvements upon this and other closures seems to be a compulsory task. In connection with the foregoing remark on **k**-space crystallization signatures, Reference [19] dealt with QHS at only one state point (γ≈0.13), obtaining some pilot results (equilateral components of the instantaneous and centroid structure factors) that showed the very slow convergence of path integral triplet calculations (also observed in Reference [33] for liquid para-hydrogen) and provided a first step for checking the methodology applied.

Furthermore, in Reference [35], helium-3 under supercritical conditions served as a prototype fluid system for analyzing quantum triplet questions in real fluids by focusing only on the instantaneous class. Three state points were studied in **r**-space and a fourth state point was in **k**-space (the diffraction conditions reached up to γ≲3.2). Path integral calculations and closures were employed and, once again, the predictive power of the intermediate closure AV3 in **r**-space was confirmed. As regards the state point studied in **k**-space, only (instantaneous) closures were employed to set a framework for further comparison [35], which was started in Reference [19] by reporting the (then available) instantaneous equilateral Fourier components determined via path integral computations. No general triplet structural quantities for the centroid class were communicated in these two previous works.

The foregoing situation demands a completion of results that, being costly, may benefit from the previously acquired data so that meaningful comparisons and interpretations can be made. In this work, PIMC simulations are performed for both systems in the canonical ensemble. The PI propagators selected are (a) for helium-3, the fourth-order propagator in Voth et al.’s form [12] (SCVJ, based on the Suzuki-Chin’s developments [98,99]), its application involving the use of Janzen–Aziz’s SAPT2 interatomic potential [100]; and (b) for QHS, Cao–Berne’s pair action [76]. Also, complementary closure calculations are reported; the following closures are utilized: Jackson–Feenberg convolution [17], Kirkwood superposition [25], the intermediate closure AV3 [34], and Denton–Ashcroft approximation in symmetrized form [47]. The results focus on salient triplet features (equilateral and isosceles) of the centroid and instantaneous structures, by covering spatial correlations in helium-3 and structure factors in helium-3 and QHS. Moreover, a number of further procedural and structural conclusions are drawn. In relation to this, the double-zero momentum transfer component $S^{\left(3\right)}(0,0)$ emerges as a potential parameter for charactering quantum fluid behaviors (e.g., far and near changes in phase). Owing to the computational cost of (and the closure basic difficulties associated with) the total thermalized-continuous linear response class [19], its related triplet computations are not attempted.

In more detail, for supercritical helium-3, the centroid triplet correlations are computed in a thorough way with path integrals and the AV3 closure; the latter keeps on showing its great usefulness under the more extreme effective conditions implied by the centroids, which mimic a fluid at a higher density than the actual one. The centroid equilateral triplet structure factor components, together with representative isosceles components, are reported and compared with the closure results fixed in this work. As regards the instantaneous case, path integral and closure representative isosceles components are also reported herein, complementing the existing equilateral information [19]. In this way, a global comparison for a real system can be made, revealing: the centroid-instantaneous differences (e.g., related to the amplitudes in general and the isosceles behavior at low angles), and the usefulness of the closures employed.

As for the QHS **k**-space investigation, the three state points studied on the crystallization line (γ≲0.13) include that at γ≈0.13 formerly considered in Reference [19]; the current gathering of statistics is conducted over longer runs. The results for this model system allow one to add valuable information to the centroid-instantaneous comparison of triplet structure factors. In relation to the quantum crystallization triplet questions, the maximal equilateral amplitudes point to the possible existence of a “constancy” for them in the centroid case that contrasts with the monotonic decay observed in the instantaneous case.

The outline of this article is as follows. Section 2 contains the basic PI theory. Section 3 describes the PI triplet centroid and instantaneous structural concepts. Section 4 focuses on the triplet closures utilized in this work and their associated theoretical features. Section 5 gives the computational details, and Section 6 the results and their discussion. Finally, Section 7 collates the conclusions of this work.

## 2. Path Integral Background

In the study of quantum monatomic fluids, one utilizes the usual statistical ensembles [1,2,26,27,28]. For constraints defining a closed system there are the canonical (N,V,T) and the isothermal–isobaric (N,p,T) ensembles, while for constraints defining an open system there is the grand canonical ensemble (μ,V,T). The variables, whose values are held fixed in each case, are the number of particles *N*, the volume V, the temperature *T*, the pressure p, and the chemical potential μ. Within the conventional statistical mechanical treatments, the atoms composing the system under study are considered structureless particles, with their spatial positions in the system being defined by those of their nuclei ${\left\{r_{j}\right\}}_{j=1,2,\ldots ,N,\ldots }.$ The latter reduction is consistent with the experimental techniques that reveal the actual existence of fluid structures [20,21,22,23,24,27], and it implies that the electronic degrees of freedom have undergone a quantum mechanical averaging process for setting an adequate potential energy interaction function $V^{\left(N\right)}\left(r_{1},r_{2},\ldots ,r_{N}\right)$ (non-collapsing and tempered [28]). Such an operation is rooted in the Born–Oppenheimer approximation [101]; model system interactions (e.g., the hard-sphere potential [27]) imply idealizations of these previous concepts. In the absence of any external field Ψ, the whole situation for a quantum system is contained essentially in the canonical density matrix given by the operator $\exp\left(-\beta H_{0}^{\left(N\right)}\right)$ [2,4,9,28]. $H_{0}^{\left(N\right)}$ stands for the isolated system Hamiltonian, which is built as $H_{0}^{\left(N\right)}=T^{\left(N\right)}+V^{\left(N\right)},$ where one includes the operators for the kinetic energy, $T^{\left(N\right)}=-\left(\hbar^{2}/2m\right)\sum_{j}\nabla_{j}^{2},$ and the potential energy, $V^{\left(N\right)}\left(r_{1},r_{2},\ldots ,r_{N}\right).$ Once the form of $H_{0}^{\left(N\right)}$ is fixed, the canonical partition function $Z^{Q}$ arises as the trace of the density matrix, $Z^{Q}(N,V,T)=Tr\left[\exp\left(-\beta H_{0}^{\left(N\right)}\right)\right]$ [2,26,28], which allows one to set the basic thermodynamic connection $A=-k_{B}TlnZ^{Q},$ where *A* stands for Helmholtz free energy. The definitions of the partition functions for the isothermal–isobaric or the grand canonical ensembles are straightforward using $Z^{Q}$ [26,27]. Within this context, recall that there is a Wick rotation contained in the formal equivalence exp(−βH)≡exp(−iHϑ/ℏ), where H stands for a time-independent Hamiltonian and ϑ for real time. The definition of an imaginary time as τ=βℏ follows from that equivalence [1,2], and τ emerges as a thermal quantum variable for the characterization of equilibrium states. Furthermore, to give operational forms to the partition functions, one uses the PI approach and obtains their proper adaptations [4,8,9,11,12,76,102,103]. To introduce the basic concepts, attention will be given to the canonical ensemble questions in this Section.

### 2.1. PI Canonical Partition Function

When using the PI formalism [2], every atom/nucleus *j* is represented by an elastic path $r_{j}\left(\tau \right)$ in imaginary time 0≤τ≤βℏ. The general form of the path integral canonical partition function of a monatomic quantum fluid, e.g., composed of helium atoms, with all the atoms in the same spin state, can be written in the canonical ensemble as follows [2]:

(1a) $$Z^{Q}\left(N,V,T\right)=Tr\left[exp(-\beta H_{0}^{\left(N\right)})\right]=\frac{1}{N!}\sum_{P}\pi_{P}\int \oint_{\begin{array}{c} r_{j}(0)=r_{j}\\ r_{j}(\beta \hbar )={Pr}_{j} \end{array}}\prod_{j=1}^{N}Dr_{j}\left(\tau \right)dr_{j}\left(0\right)\times \;exp\left[-\frac{1}{\hbar }\int_{0}^{\beta \hbar }\left(\sum_{j=1}^{N}\frac{m{\left(\dot{r_{j}}\right)}^{2}}{2}+V^{\left(N\right)}\left(r_{1}\left(\tau \right),r_{2}\left(\tau \right),\ldots ,r_{N}\left(\tau \right)\right)\right)d\tau \right].$$

The symbol P runs over the N! permutations among the N indistinguishable atoms, and $\pi_{P}$ is the sign of the permutation P (i.e., +1 for every boson permutation; ±1 for fermion permutations that, depending on the parity, is +1 for even P or −1 for odd P). In Equation (1a), the **r**-space integrations cover all the configuration space associated with the particle paths ${\left\{r_{j}\left(\tau \right)\right\}}_{j=1,2,\ldots ,N}$, and the symmetry constraints are denoted by the conditions at τ=0 and τ=βℏ, which imply that the particle paths may interlink for P≠ Identity in a large variety of ways. The exp-factor contains the action in imaginary time, where $\dot{r_{j}\;}$ stands for the derivative with respect to τ, and $V^{\left(N\right)}$ is the potential energy function acting at equal-τ “instants”. Equivalently, $Z^{Q}$ can also be cast in the coordinate representation as follows [2,4,9]:

(1b) $$Z^{Q}\left(N,V,T\right)=Tr\left\{exp(-\beta H_{0}^{\left(N\right)})\right\}=\frac{1}{N!}\sum_{P}\pi_{P}\int dr^{N}\left\langle r^{N}|exp(-\beta H_{0}^{\left(N\right)})|{Pr}^{N}\right\rangle ,$$

where $dr^{N}=dr_{1}dr_{2}\ldots dr_{N}$ is the 3N− dimensional volume element of the configurational space of the N actual atoms, $|r^{N}\rangle$ is the ket of position states $|r_{1}{,r}_{2},\ldots ,r_{N}\rangle$, and the permutations act on the particle position states as ${|Pr}^{N}\rangle =$ $|Pr_{1}{,Pr}_{2},\ldots ,{Pr}_{N}\rangle .$ At sufficiently high temperature, only the identity permutation contributes to effectively shape Equation (1); the BE or FD quantum statistics features can be neglected, and both exchange situations lead to the same general result in the form of “distinguishable” delocalized atoms (this is nicely illustrated by helium systems [9,96]). Such a situation is that of thermal quantum diffraction/dispersion effects, for which the PI canonical partition function reads as follows [2,4]:

(2a) $$Z^{D}\left(N,V,T\right)=Tr{\left\{exp(-\beta H_{0}^{\left(N\right)})\right\}}_{D}=\frac{1}{N!}\int \oint_{r_{j}(0)=r_{j}(\beta \hbar )}\prod_{j=1}^{N}Dr_{j}\left(\tau \right)dr_{j}\left(0\right)\times \;exp\left[-\frac{1}{\hbar }\int_{0}^{\beta \hbar }\left(\sum_{j=1}^{N}\frac{m{\left(\dot{r_{j}}\right)}^{2}}{2}+V^{\left(N\right)}\left(r_{1}\left(\tau \right),r_{2}\left(\tau \right),\ldots ,r_{N}\left(\tau \right)\right)\right)d\tau \right],$$

where every atom *j* is represented by an elastic closed path $r_{j}\left(\tau \right)$ in imaginary time, such that $r_{j}\left(0\right)=r_{j}\left(\beta \hbar \right).$ In the coordinate representation, Equation (2a) reads as follows [4,9]:

(2b) $$Z^{D}\left(N,V,T\right)=Tr{\left\{exp(-\beta H_{0}^{\left(N\right)})\right\}}_{D}=\frac{1}{N!}\int dr^{N}\left\langle r^{N}|exp(-\beta H_{0}^{\left(N\right)})|r^{N}\right\rangle .$$

In Equation (2), although only the identity permutation is retained and the atoms can be “numbered”, the presence of N! as the remaining factor of the true indistinguishability among the atoms is to be noticed; the presence of ℏ, as a factor necessary to guarantee the correct dimensionality, is also to be noticed. (Both N! and ℏ are indispensable for the formulation of the classical partition function [2]). In general, for many-body system studies, Equations (1b) and (2b) are the practical forms of the partition function that serve as starting points to develop the PI-discretized numerical approaches [4,9,12,76].

In this work, attention is focused on Equation (2b) and its connections with the incorporation of weak external fields $\Psi^{\left(N\right)}$ acting on the atoms/nuclei/particles of the quantum fluid. The fields define the three classes of equilibrium structures $\left\{g_{n},S^{\left(n\right)}\right\}$ in a fluid under quantum diffraction conditions, with linear response theory being instrumental in the corresponding derivations. In this context, the specific nature of the field and its interactions with the atoms/nuclei/particles are determinant, which contrasts with its classical counterpart [19,27,32,77,78,79].

Based on this connection, some cautionary remarks are to be made. The discussion in this Section considers the path integral case involving weak continuous fields, which is a situation amenable to classical-like treatments and allows one to introduce most of the basic concepts. It so happens that the total thermalized-continuous linear response (TLRn) and centroid (CMn) classes are directly linked to the following developments [14,19,32]. However, the instantaneous class (ETn) escapes such direct treatment based on continuous fields, owing to the localizing fields involved in its definition. Despite the previous remark, Equation (2b) serves equally well the purposes of formulating the instantaneous structural functions at any *n*-level, because the underlying arguments involved are also associated with linear response theory [9,19,27]. The structural facts relevant to this work (CMn and ETn) are considered in the next Section.

### 2.2. The Action of Weak External Continuous Fields

In the presence of a weak external continuous field Ψ, acting as $\Psi^{\left(N\right)}=\sum_{j=1}^{N}\Psi \left(r_{j}\right),$ the canonical partition function for diffraction effects can be cast in the coordinate representation as follows [14]:

(3) $$Z^{D}\left(N,V,T;\Psi \right)=Tr{\left\{exp(-\beta {(H}_{0}^{\left(N\right)}+\Psi^{\left(N\right)}))\right\}}_{D}=\;\;\;\;\;\;\;\;\;\;\;\;\;\;\;\;\;\;\;\;\;\;\;\;\;\;\;\;\;\;\;\;\;\;\;\;\;\;\;\;\;\;\frac{1}{N!}\int dr^{N}\left\langle r^{N}|exp(-\beta {(H}_{0}^{\left(N\right)}+\Psi^{\left(N\right)}))|r^{N}\right\rangle .$$

The operators $H_{0}^{\left(N\right)}$ and ${H^{\left(N\right)}=H}_{0}^{\left(N\right)}+\Psi^{\left(N\right)}$ are assumed to be self-adjoint, having complete sets of eigenvectors and eigenvalues. (To generalize the right-hand side of Equation (2a) by including Ψ, the action integral should contain the term $\Psi^{\left(N\right)}\left(r_{1}\left(\tau \right),r_{2}\left(\tau \right),\ldots ,r_{N}\left(\tau \right)\right)).$ By applying well-established mathematical procedures [9,12,14,104] (see Appendix A.1), one obtains the PI-discretized approximation $Z_{PI}^{D}$ for the canonical partition function that reads as follows [14]:

(4) $$Z^{D}\left(N,V,T;\Psi \right)\approx Z_{PI}^{D}\left(N,V,T;\Psi \right)=\;\frac{1}{N!}\int \prod_{t^{*}=1}^{X^{\prime }}dr^{N,t*}\left\langle r^{N,t^{*}}|exp(-\beta H_{0}^{\left(N\right)}/X)|r^{N,t^{*}+1}\right\rangle \times \exp\left(-\frac{\beta }{X}\sum_{j=1}^{N}\sum_{t^{*}=1}^{X}\Psi (r_{j}^{t^{*}})\right),$$

where $t^{*}=1,2,\ldots ,X,$ the primed symbol $X^{\prime }$ implies the cyclic property $t^{*}+1=X+1\equiv 1$ for the $t^{*}—$product, every particle (*j*) path is discretized into *X* stages (or “instants”) in imaginary time βℏ, the matrix elements $\left\langle r^{N,t^{*}}|exp(-\beta H_{0}^{\left(N\right)}/X)|r^{N,t^{*}+1}\right\rangle$ are the thermal propagators, and the integrations cover the whole configuration space of the marked stages $dr^{N,t*}={dr}_{1}^{t^{*}}dr_{2}^{t^{*}},\ldots ,dr_{N-1}^{t^{*}}dr_{N}^{t^{*}}.$ Thus, one obtains the path integral image of necklaces *j* and their structurally significant beads $r_{j}^{t^{*}}\;$(in Equation (4), note the action of the field as the *X*-average of the field–bead couplings). As discussed below, this image may not be the final one.

$Z_{PI}^{D}$ is accurate up to terms $O(X^{-2})$. In the limit X→∞, one retrieves the actual $Z^{D}(\Psi ),$ since the operators $H_{0}^{\left(N\right)}$ and $\Psi^{\left(N\right)}$ are self-adjoint and make sense separately, as required by Trotter’s analysis [3,9] (the same applies to $T^{\left(N\right)}$ and $V^{\left(N\right)}$ regarding questions that involve the isolated $H_{0}^{\left(N\right)}$ operator [9]). Given that the propagators contained in Equation (4) are necessarily nonnegative everywhere in the configuration space corresponding to the set of variables $\left\{r^{N,t^{*}}\right\}$, it is easy to identify the underlying probability density of this formulation. Therefore, classical-like statistical developments and calculations involving $Z_{PI}^{D}$ can be performed, and X represents a compromise between statistical convergence and theoretical accuracy (i.e., X→∞) [3], which means that X can be properly optimized for the problem under study. Remarkably, linear response theory utilizes the structural functions of the isolated-from-Ψ fluid, that is, those functions derived from the consideration of $H^{\left(N\right)}=H_{0}^{\left(N\right)}$ alone. Hence, questions on the accuracy of the structures obtained through the use of Equation (4), related to the number of “instants” X, are merely formal, because one can optimize X by analyzing just the isolated-from-Ψ fluid structures.

### 2.3. Propagators for the PI-Discretized Canonical Partition Function

At this point, it is convenient to discuss the options for the general propagator $\left\langle r^{N,t^{*}}|exp(-\beta H_{0}^{\left(N\right)}/X)|r^{N,t^{*}+1}\right\rangle$ in the absence of Ψ, which will give final forms to $Z_{PI}^{D}$. For structural studies, there are three main types of propagators: primitive [4,5,6,7,8,9,14], pair actions [9,76], and the fourth-order SCVJ [12,98,99]. (See also another fourth-order propagator version in Reference [105]). Two main interrelated facts take part in their derivations. First, there is the form in which the imaginary time step is chosen. Thus, if β is divided into X=P equally spaced intervals, with the step-unit set to β/P, one will deal with the primitive or a pair action propagator. However, if a double step 2β/P is selected, making P be an even positive integer, i.e., X=P/2, one can deal with the SCVJ propagator. In the end, for practical and consistency reasons, the final number of intervals will be *P*, regardless of the type of propagator selected, although the roles of the interval ends may not be equivalent in the final description achieved (e.g., the SCVJ case). Second, there are different choices to deal with the noncommutativity between $T^{\left(N\right)}$ and $V^{\left(N\right)}.$ These choices are in the roots of the picture of N necklaces with P beads apiece for the discretized closed paths in imaginary time, $r_{j}(t^{*}$), of the particles: such N×P picture is common to the three types of propagators considered. In this connection, for a sufficiently large P, the three propagators converge to the same final description; thus, a critical issue in PI calculations is the distinct statistical convergence rates that, depending on the optimal *P*, can be achieved with each type of propagator [9,12,14,76].

Although for specific details the reader is referred to the References quoted above, for the current purposes, it is worth stating that the general form of $Z_{PI}^{D}$ can be cast as follows [9,12,14]:

(5) $$Z_{PI}^{D}\left(N,V,T;\Psi \right)=\;\frac{1}{N!}{\left(\frac{mP}{2\pi \beta \hbar^{2}}\right)}^{3NP/2}\int \prod_{t=1}^{P}dr^{N,t}\times exp\left(-\beta W_{NP}(r_{1}^{1},r_{1}^{2},\ldots ,\;r_{1}^{P},\ldots ,r_{N}^{P};\beta ,\hbar ,m\right))\times \exp\left(-\frac{\beta }{X}\sum_{j=1}^{N}\sum_{t^{*}=1}^{X}\Psi (r_{j}^{t^{*}})\right),$$

where X=P or P/2 and, for notational simplicity, two symbols to denote the beads, *t* and $t^{*},$ are employed. The relations between the bead sets {t=1,2,…,P} and $\left\{t^{*}=1,2,\ldots ,X\right\}$ are explained below (the $t^{*}$ elements coincide with choices extracted from the t set) [14].

In Equation (5), the integrations cover the whole 3NP-dimensional configurational space associated with the N closed necklaces, j=1,2,…,N; each necklace is composed of P beads, t=1,2,…,P. $W_{NP}$ contains the specific connections/interactions among all the beads in the sample, thereby being an “effective potential” for the resulting classical-like set of N×P beads. Thus, one finds the so-called (semi) classical isomorphism [4], in which the $W_{NP}$ form that contains the bead interactions depends on the propagator selected and, also, on β,m, and ℏ. While for the primitive and pair action propagators, the situation is straightforward (X=P), and the SCVJ derivation doubles necessarily the initial number X of instants, $t^{*}=1,2,\ldots ,X=P/2,$ by adding P/2 intermediate instants to the initially closed *X*-trajectory [12]. Therefore, the complete SCVJ bead set is renumbered according to the total number of beads P, t=1,2,3,…,P−1,P, and the odd-numbered ones are associated with the field–bead interactions, as stated in Equations (4) and (5). Linear response theory [14,77] uses such interactions in its derivations and, therefore, one can anticipate that the significant beads for the definitions of structures will be as follows: (a) for the primitive and pair action propagators, X=P and $t^{*}=t=1,2,3,\ldots ,P,$ with all the beads being significant and equivalent, and (b) for SCVJ, X=P/2 and t=1,2,3,…,P, with the odd-numbered beads playing the role of the $t^{*}$ initial ones, $t^{*}\equiv t=1,3,5,\ldots ,P-1$, with these being the structurally significant ones and also equivalent among themselves. (Equivalent means that the mathematical treatment is the same for every related bead in the sample; the even-numbered beads are also equivalent among themselves).

It may be worthwhile to highlight some facts contained in the foregoing discussion. Each closed path contains X marked imaginary time instants (X=P or X=P/2), such that conventionally, $t^{*}=1$ corresponds to $\tau_{1}=0\equiv \beta \hbar ,$ $t^{*}=2$ to $\tau_{2}=\frac{\beta \hbar }{X},$ $t^{*}=3$ to $\tau_{3}=\frac{2\beta \hbar }{X},$…, and $t^{*}=X$ to $\tau_{X}=\frac{(X-1)\beta \hbar }{X}$. The continuous description is retrieved in the limit X→∞, which may seem obvious but runs deeper than one might think at first sight, as no errors creep in the process (i.e., Trotter’s theoretical accuracy) [3,9]. Moreover, note that the taking of the imaginary time origin at $\tau_{1}=0$ is nothing special; it could have been taken at any other $\tau_{n}$ instant of the *X*-sequence, since translational τ—invariance must hold. In this regard, the only requirement is that once the origin is chosen, it must be applied to every atom/nucleus/particle path in the system for consistency reasons [9]. The imaginary time evolution is periodic in that 0≡βℏ, as is customary in Fourier analysis (this does not mean that β=0 whatsoever!). Finally, the recipe for structural studies: all the beads are significant in the primitive and pair action cases, although only the odd-numbered beads are significant in the SCVJ case.

For the reader’s convenience, the explicit forms of the effective potential $W_{NP}$ dealt with in this article are worth giving. The optimal *P* discretization is assumed in each case. For helium-3, the SCVJ propagator leads to [12]:

(6) $$W_{NP}^{SCVJ}=\frac{(m_{3_{\mathrm{He}}})P}{2\beta^{2}\hbar^{2}}\sum_{j=1}^{N}\sum_{t=1}^{P^{\prime }}{\left(r_{j}^{t}{-r}_{j}^{t+1}\right)}^{2}+\frac{2}{3P}\sum_{j<l}\left\{\sum_{\begin{array}{c} t-odd\\ 1,3,5,\ldots ,P-1 \end{array}}u\left(r_{jl}^{t}\right)+2\sum_{\begin{array}{c} t-even\\ 2,4,6,\ldots ,P \end{array}}u\left(r_{jl}^{t}\right)\right\}+\;\frac{\beta^{2}\hbar^{2}}{9(m_{3_{\mathrm{He}}})P^{3}}\sum_{j=1}^{N}\left\{\alpha \sum_{\begin{array}{c} t-odd\\ 1,3,5,\ldots ,P-1 \end{array}}{\left(\sum_{l\neq j}\frac{\partial u\left(r_{jl}^{t}\right)}{\partial r_{jl}^{t}}\eta_{jl}^{t}\right)}^{2}+(1-\alpha )\sum_{\begin{array}{c} t-even\\ 2,4,6,\ldots ,P \end{array}}{\left(\sum_{l\neq j}\frac{\partial u\left(r_{jl}^{t}\right)}{\partial r_{jl}^{t}}\eta_{jl}^{t}\right)}^{2}\right\},$$

where $m=m_{3_{He}}$ is the atom mass, use is made of the pairwise interaction approach $V^{\left(N\right)}=\sum_{j<l}u\left(r_{jl}\right)$ involving the pair potentials $u\left(r_{jl}\right)$, $r_{jl}^{t}=\left|r_{j}^{t}-r_{l}^{t}\right|,$ the primed symbol $P^{\prime }$ denotes the cyclic property t+1=P+1≡1, the parameter α is a real number in [0,1], and $\eta_{jl}^{t}$ is the unit vector $\frac{r_{jl}^{t}}{r_{jl}^{t}}.$ Note that the interactions between beads occur if and only if the beads share the same *t*-label, and that a recommended value for α is α=1/3 [12]. Furthermore, the nonequivalence between even- and odd-numbered beads is apparent; it influences not only the structural calculations but also the thermodynamic ones, although in quite different practical forms (e.g., in thermodynamic evaluations using α=1/3, all the beads play a role) [12,14,96,97]. The SCVJ propagator is accurate up to $O\left(P^{-5}\right)$ and its associated partition function is up to $O\left(P^{-4}\right)$. For the quantum hard-sphere fluid, the effective potential $W_{NP}$ is built with the Cao–Berne propagator (CBHSP) [76] that, for hard spheres with mass $m=m_{HS}$ and diameter σ, can be cast as follows [81,82,97]:

(7) $$W_{NP}^{CBHSP}=\frac{{(m}_{HS})P}{2\beta^{2}\hbar^{2}}\sum_{j=1}^{N}\sum_{t=1}^{P^{\prime }}{\left(r_{j}^{t}{-r}_{j}^{t+1}\right)}^{2}+\frac{1}{P}\sum_{j<l}\sum_{t=1}^{P}u_{HS}\left(r_{jl}^{t}\right)-\;\frac{1}{\beta }ln\prod_{j<l}\prod_{t=1}^{P^{\prime }}\left\{1-\frac{\sigma (r_{jl}^{t}+r_{jl}^{t+1}-\sigma )}{r_{jl}^{t}\;r_{jl}^{t+1}}exp\left(-\frac{{(m}_{HS})P}{2\beta \hbar^{2}}{(r}_{jl}^{t}-\sigma )(r_{jl}^{t+1}-\sigma )(1+cos{\;\varphi }_{jl}^{t,t+1})\right)\right\},$$

where, once again, the cyclic property implied by $P^{\prime }$ applies to the corresponding sum and product that run over *t*. The pairwise approach for the interactions between equal-*t* beads is used; $u_{HS}$ is the usual hard-sphere potential that vanishes for $r_{jl}^{t}>\sigma$ and becomes infinity for $r_{jl}^{t}<\sigma ,$ and ${\;\varphi }_{jl}^{t,t+1}$ is the angle defined by $r_{jl}^{t}$ and $r_{jl}^{t+1}.$ The equivalence among all the beads in the sample is clear in this case. Also, there are no specific rules for the accuracy of pair action propagators; their effectiveness is to be checked by increasing *P*, but they are extremely efficient in reducing the optimal number of beads [9,70,71,76,106,107,108]. Equations (6) and (7) illustrate the analogies and differences between the effective potentials $W_{NP}$ for both types of systems. Both possess a formally identical first term on the right-hand side that contains the image of *P*-membered closed necklaces (i.e., free-particle contributions [2,4]). However, the rest of the terms clearly differ from one another, owing to (a) the characteristics of the interparticle potentials involved in their constructions, which bring about the appearance of forces in SCVJ [12] and of kinetic correlation effects in CBHSP (the contributions from adjacent beads in different necklaces) [97], and (b) the bead symmetry and asymmetry present in CBHSP and SCVJ, respectively.

## 3. Quantum Fluid Structures

The whole set of equilibrium structures of a monatomic fluid in the thermal quantum diffraction regime can be deduced from the basic form given in Equation (5). In achieving this task, the related developments are to be complemented with the linear response considerations of external weak fields Ψ acting on the fluid [14,19,32,78,79]. Thus, one finds the following classes and associations: (a) total thermalized-continuous linear response TLRn, associated with continuous external fields $\Psi^{\left(N\right)}=\sum_{j}{\Psi (r}_{j});$ (b) centroids CMn, as a particular case of TLRn, since its corresponding continuous field acts as $\Psi_{F}^{\left(N\right)}=\sum_{j}{f\cdot r}_{j},\;$ where **f** is a constant strength; and (c) instantaneous ETn, associated with singular fields that cause the localization of the thermally delocalized quantum atoms (nuclei) in the fluid sample, i.e., the fields used in elastic X-ray diffraction or in the inelastic scattering of neutrons [20,21,22,24], for which collision processes are involved [27,79]. (As stressed earlier, zero-spin-atom BE fluids also follow the same systematics [19,78,79]).

Remarkably, the classes CMn and ETn keep a classical-like pattern in that the thermal quantum delocalization of the atoms (nuclei), although accounted for in their developments at every structural *n*-level, is not obviously patent in the resulting analytic equations for their structure factors [9,19]. In sharp contrast, the class TLRn includes nontrivial particle self-correlations in the formulations at every *n*-level [14,19,32].

The analysis involving fields completes the standard probabilistic method for the definition of structures [9,12,13,26]. For conceptual reasons [26,27,28,37,45], these quantum developments can be extended fully if they are carried out in the grand canonical ensemble [14,19,30,32], although the use of the canonical ensemble also serves the primary purpose of establishing basic formal definitions of the structures [26,27,28]. In this connection, it is worthwhile to stress that, although the parallel reasoning employed in the classical domain is based on functional calculus operating with a general field Ψ, and is an excellent guide [27,37,45], the quantum complexity does not allow one to follow such a procedure in its entirety [19]. The specific nature of Ψ may make certain classical-like manipulations either meaningful or void in the quantum domain. Moreover, some special mathematical objects that are perfectly defined in the classical domain, as associated with the grand canonical ensemble (i.e., the direct correlation functions $c_{Cn})$ [37,45], may not be defined in an exact manner for every quantum structural case [19,32]. Given that, for computational reasons, this work will be focused on CM3 and ET3, attention to their main theoretical features is given in this Section.

### 3.1. The Centroid Structures

#### 3.1.1. Opening Centroid Facts

The centroid concept in quantum statistical mechanics arose within Feynman’s path integral formulation (PI) of the equilibrium behavior of many-body monatomic systems at nonzero temperatures [1,2]. For the original centroid definition to be made [1], thermal quantum diffraction effects were required to completely dominate the system behavior (generalizations of this concept came later in connection with the PI simulation of quantum exchange regimes [87,88,89]). The centroid associated with atom *j* is defined as the “center of mass” $R_{CM,j}$ of its representative closed path $r_{j}\left(\tau \right)$ that, for this purpose, is always considered to have a uniform “mass-density” along its contour. Thus, the centroid concept can be formulated per atom *j* as follows [1,2]:

(8) $$R_{CM,j}=\frac{1}{\beta \hbar }\int_{0}^{\beta \hbar }r_{j}\left(\tau \right)d\tau ;\;\;j=1,2,\ldots ,N,\ldots .$$

The same definition may be applied to every delocalized particle composing neat many-body systems, so long as they may be described by one-site models (e.g., liquid deuterium [109], liquid para-H_2_ [97,110,111,112], liquid N_2_ [113], the hard-sphere fluid or solid [29,81,82,97], etc.).

Application of a variational principle leads to approximate (semiclassical) forms of the partition function in terms of the foregoing centroid variables [1,2,114,115]. The main result of these approximations is contained in the centroid-effective interparticle potentials $w_{F}\left(R_{jl};\beta ,m,\hbar \right)$ that shape the variational semiclassical partition functions (intercentroid distance $R_{jl}=\left|R_{CM,j}-R_{CM,l}\right|).$ The $w_{F}$ potentials correct the (usual) interatomic potential energies u(r) but differ dramatically from them in that they depend on the parameters β,m, and ℏ. There is, obviously, formal equivalence among the centroids that can be defined in this simplified way to model a given monatomic fluid. However, it is worth realizing that these objects are not representations of the true particles/atoms of the fluid [14]. As stressed earlier, centroids mimic a fluid at a higher density than the actual one, and the conclusions drawn from their only use do not necessarily apply to the properties of the quantum system under study [12,13,14,80,85,90,94,114,115,116,117,118].

The best illustration of the foregoing fact is, perhaps, given by the centroid structures, which can be directly calculated through simulations based on the centroid-effective partition functions in classical-like ways [14,84,85,117]. The determination of the actual particle correlation structures does need special convolutions [84,85,117]; the latter involve the joint consideration of the centroid structures and the particle thermal quantum packets (i.e., representations of a delocalized particle around its centroid, which may be nontrivial Gaussians), and only yield approximations to the true path integral TLRn functions [14,85]. These PI centroid-based approaches are far more useful than the well-known Wigner–Kirkwood expansion [67,119,120], even when the latter is extended to highly sophisticated forms [121,122,123]. Nonetheless, the thermodynamic and structural predictive powers of the $w_{F}$ effective potential pictures are limited: they cannot yield complete structural results, nor lead to accurate pictures of the real fluid under increasing quantum effects [14,85].

Despite the incompleteness of the centroid-effective approximations, the usefulness of the centroid concept as seen from the full PI perspective exceeds expectations [12,19,34,78,90,94,112] (see below). The starting point is the discretized version of the PI formulation, Equation (5), which involves a faithful probability density (i.e., nonnegative everywhere). Note that no variational approximation is involved here and, therefore, this “exact” realization of the centroid concept is not the same as that arising from the $w_{F}$ schemes [85]. Through PIMC and PIMD simulations, one can gather statistics related to the *n*-body general structural quantities (correlation functions $g_{n}/G_{n}$ and structure factors $S^{\left(n\right)}$), in particular the centroid structures [19,32,33,34,35,78,97]. The grand canonical ensemble derivations for the centroid CMn class need to consider a field $\Psi_{F}$ of constant strength **f** [19,32,78,80,94], and its main features are given below.

#### 3.1.2. PI Centroid Formulations

The PI grand canonical partition function in the presence of $\Psi_{F}$ can be written as follows:

(9) $$\Xi_{PI}^{D}\left(\mu ,V,T;\Psi_{F}\right)=\sum_{N\geq 0}exp\left(\beta \mu N\right)\;Z_{PI}^{D}\left(N,V,T;\Psi_{F}^{\left(N\right)}\right).$$

For the SCVJ and CBHSP frameworks, the centroid variables are given by the following [12,14]:

(10a) $$R_{CM,j}=\frac{2}{P}\sum_{t=1,3,\ldots ,P-1}r_{j}^{t},\;\mathrm{for SCVJ},$$

(10b) $$R_{CM,j}=\frac{1}{P}\sum_{t=1,2,\ldots ,P}r_{j}^{t},\;\mathrm{for CBHSP},$$

where each definition can be viewed as the proper discretization of Equation (8). Therefore, the general form of the partition function can be reformulated as follows [19,30,78]:

(11) $$\Xi_{PI}^{D}\left(\Psi_{F}\right)=\sum_{N\geq 0}\frac{z^{N}}{N!}\int \left\{\prod_{j=1}^{N}dR_{j}\right\}\int \prod_{t=1}^{P}dr^{N,t}\times \left\{\prod_{j=1}^{N}\delta \left(R_{j}-R_{CM,j}\right)\right\}\times \;exp\left(-\beta W_{NP}\right)\times \exp\left(-\beta \sum_{j=1}^{N}\Psi_{F}(R_{j})\right).$$

In Equation (11), $z=\exp\left(\beta \mu \right){\left(\sqrt{P}\lambda_{B}^{-1}\right)}^{3P},$ and $W_{NP}$ stands for the corresponding PI effective potential. The foregoing form of the partition function is akin to a (semi-)classical partition function, and it is ready to carry out the functional derivatives with respect to the field, i.e., $\frac{\delta^{n}ln\Xi_{PI}^{D}\left(\Psi_{F}\right)}{\delta \Psi_{F}(R_{1})\delta \Psi_{F}\left(R_{2}\right){\ldots \delta \Psi }_{F}\left(R_{n}\right)},$ by applying the standard classical procedures [27,28,45,77]. Accordingly, the structural results for centroids are of a clear classical-like nature [19,30,78]. Hereafter, the grand canonical ensemble averages at zero field $\left(\Psi_{F}=0\right)$ will be denoted by 〈…〉, and those in the canonical ensemble by ${\langle \ldots \rangle }_{Z_{PI}^{D}}.$

The first three functional derivatives of Equation (11) with respect to $\Psi_{F}(R_{j})$ lead to the generalized centroid structural functions $\Gamma_{CMn}(\Psi_{F})$ up to the triplet level (n=1,2,3), as shown in References [19,32]. By applying linear response arguments, one takes $\Psi_{F}=0$ and the usual $g_{CM2}(R)$ and $g_{CM3}(R_{12},R_{13},R_{23})$ correlation functions can be identified as constituent parts of $\Gamma_{CM2}(R_{1},R_{2};\Psi_{F}=0)$ and $\Gamma_{CM3}(R_{1},R_{2},R_{3};\Psi_{F}=0).$ These formulations are far from trivial and increase in complexity with increasing order *n*. The structure factors at the pair and the triplet levels, $S_{CM}^{\left(2\right)}\left(k\right)$ and $S_{CM}^{\left(3\right)}\left(k_{1},k_{2}\right),$ arise essentially from Fourier transforming $\Gamma_{CM2}(\Psi_{F}=0)$ and $\Gamma_{CM3}(\Psi_{F}=0),$ respectively. For a quick explanation of these facts, see Appendix A.2.

In this work, the centroid spatial functions investigated, $g_{CM2}(R)$ and $g_{CM3}\left(R_{12},R_{13},R_{23}\right),$ are analogous to the usual two- and three-body correlation functions in the theory of classical homogeneous and isotropic fluids [26,27,28]. Their definitions are given by the following ensemble averages involving Equation (11) at $\Psi_{F}=0:$

(12) $${\rho_{N}^{2}g}_{CM2}\left(R_{12}\right)=\langle \sum_{j\neq l}\delta \left(R_{CM,j}-q_{1}\right)\delta \left(R_{CM,l}-q_{2}\right)\rangle ,$$

(13) $${\rho_{N}^{3}g}_{CM3}\left(R_{12},R_{13},R_{23}\right)=\langle \sum_{j\neq l\neq m\neq j}\delta \left(R_{CM,j}-q_{1}\right)\delta \left(R_{CM,l}-q_{2}\right)\delta \left(R_{CM,m}-q_{3}\right)\rangle ,$$

where the bulk (number) density is denoted by $\rho_{N}=\langle N\rangle /V$, and the generic intercentroid distances are defined in terms of auxiliary **q** variables, i.e., $R_{ab}=\left|q_{a}-q_{b}\right|.$ These spatial averages can be determined via PI simulation by following procedures parallel to those applied in the study of classical monatomic fluids [29,30,31,32,33,34,41,46,48].

The complete formulations of the static structure factors, the pair $S_{CM}^{\left(2\right)}(k)$ and the triplet $S_{CM}^{\left(3\right)}\left(k_{1},k_{2}\right),$ read as follows [19]:

(14) $$S_{CM}^{\left(2\right)}\left(k\right)=1+\rho_{N}\int dR\exp\left(ik\cdot R\right)h_{CM2}\left(R\right)=1+\rho_{N}h_{CM}^{\left(2\right)}\left(k\right),$$

(15) $$S_{CM}^{\left(3\right)}\left(k_{1},k_{2}\right)=1+\rho_{N}\left[h_{CM}^{\left(2\right)}\left(k_{1}\right)+h_{CM}^{\left(2\right)}\left(k_{2}\right)+h_{CM}^{\left(2\right)}\left(k_{1}+k_{2}\right)\right]+\;\;\rho_{N}^{2}[g_{CM}^{\left(3\right)}\left(k_{1},k_{2}\right)-h_{CM}^{\left(2\right)}\left(k_{1}\right){\left(2\pi \right)}^{3}\delta \left(k_{1}+k_{2}\right)-h_{CM}^{\left(2\right)}\left(k_{2}\right){\left(2\pi \right)}^{3}\delta \left(k_{1}\right)\;-h_{CM}^{\left(2\right)}\left(k_{1}\right){\left(2\pi \right)}^{3}\delta \left(k_{2}\right)-{\left(2\pi \right)}^{3}\delta \left(k_{1}\right){\left(2\pi \right)}^{3}\delta \left(k_{2}\right)],$$

where the total correlation function $h_{CM2}\left(R\right)=g_{CM2}\left(R\right)-1$ and its Fourier transform $h_{CM}^{\left(2\right)}\left(k\right)$ are employed. In the foregoing formulas, the **k** wave vectors define the momentum transfers from the field to the fluid (i.e., ℏk). The operative expressions take the forms $S_{CM}^{\left(2\right)}\left(k\right)$ and ${\;S}_{CM}^{\left(3\right)}\left(k_{1},k_{2},cos(k_{1},k_{2}\right))\equiv S_{CM}^{\left(3\right)}\left(k_{1},k_{2},\phi \right),$ in which a modulus-variable k=|k| denotes the corresponding wavenumber and ϕ is the angle between the wave vectors $k_{1}$ and $k_{2}.$ However, from the PI computational standpoint, the centroid pair and triplet structure factors are fixed through the ensemble averages [18,19,32,96]:

(16) $$S_{CM}^{\left(2\right)}\left(k\right)\to \frac{1}{\langle N\rangle }\langle \sum_{j=1}^{N}\sum_{l=1}^{N}exp\left(ik\cdot \left[R_{CM,j}-R_{CM,l}\right]\right)\rangle ,$$

(17) $$S_{CM}^{\left(3\right)}\left(k_{1},k_{2}\right)\to \frac{1}{\langle N\rangle }\langle \sum_{j=1}^{N}\sum_{l=1}^{N}\sum_{m=1}^{N}exp\left[i\left(k_{1}\cdot \left(R_{CM,j}-R_{CM,m}\right)+k_{2}\cdot \left(R_{CM,l}-R_{CM,m}\right)\right)\right]\rangle ,$$

where the reader’s attention should be drawn to the fact that the δ(k)-evaluations at $k=0,k_{1}=0,k_{2}=0,$ and $k_{1}+k_{2}=0$ are avoided, as they are not directly accessible in simulation work [27,46].

From a practical standpoint, the correlation function and structure factor calculations are more affordable using the canonical ensemble (number density $\rho_{N}=N/V)$, involving a sample size $N_{S}$ in a box of volume $V_{S}$ at temperature *T*. In relation to this, the corresponding averages for the centroid structures given above, Equations (12)–(17), only need to reflect the changes associated with the reduction $\langle \ldots \rangle \to {\langle \ldots \rangle }_{Z_{PI}^{D}}$; for example, the triplet Equation (17) in the canonical ensemble reads as follows:

(18) $$S_{CM}^{\left(3\right)}\left(k_{1},k_{2}\right)\to \frac{1}{N}{\langle \sum_{j=1}^{N}\sum_{l=1}^{N}\sum_{m=1}^{N}exp\left[i\left(k_{1}\cdot \left(R_{CM,j}-R_{CM,m}\right)+k_{2}\cdot \left(R_{CM,l}-R_{CM,m}\right)\right)\right]\rangle }_{Z_{PI}^{D}}.$$

As for the zero-wavenumber situations (k→0), costly PI extrapolation procedures must be employed to obtain approximate answers: by increasing consistently $N_{S}$ and $V_{S},$ so as to keep the number density $\rho_{N}=N_{S}/V_{S}$ constant [46], lower *k* can be reached and extrapolations to zero wavenumbers may be obtained.

Also, note that the centroid structures are intermediate quantities, since the application of a weak field of constant strength would lead to the TLR2 response function as the, in principle, measurable object [19,30,32]. Centroids as such do not couple physically with external fields, actual particles do.

#### 3.1.3. The Exact Centroid OZn Framework

In the quantum diffraction regime, an important point regarding PI centroids, Equation (10), is that they behave according to the same rules for structures applicable to classical monatomic fluids at equilibrium [27,40,45]. This is of great practical importance when addressing **k**-space questions. In fact, it has been demonstrated [19] that the (hierarchies of) centroid direct correlation functions and structure factors $\left\{c_{CMn},S_{CM}^{\left(n\right)}\right\}$ can be defined in the same fashion as in the classical domain. Therefore, the underlying classical Ornstein–Zernike framework (OZn) [37,38,40,45,124,125,126,127,128], which is consistently defined in the grand canonical ensemble, can be safely applied to the PI centroid correlations and their structure factors. Hence, one may deal with the centroid zero-wavevector problems considered above in an essentially “exact” manner. In particular, at the pair OZ2 and triplet OZ3 levels, in the absence of the external field ($\Psi_{F}=0),$ one can write rigorously the following basic equations for PI centroids [19,30]:

(19) $$h_{CM2}\left(R_{12}\right)=c_{CM2}(R_{12})+\rho_{N}\int dR_{3}c_{CM2}\left(R_{13}\right)h_{CM2}\left(R_{23}\right),$$

(20a) $${\left(\frac{\partial c_{CM2}(R_{12};\rho_{N})}{\partial \rho_{N}}\right)}_{T}=\int dR_{3}c_{CM3}\left(R_{1},R_{2},R_{3};\rho_{N}\right),$$

(20b) $${\left(\frac{\partial c_{CM}^{\left(2\right)}(k_{1};\rho_{N})}{\partial \rho_{N}}\right)}_{T}=c_{CM}^{\left(3\right)}\left(k_{1},k_{2}=0;\rho_{N}\right),$$

(21) $$S_{CM}^{\left(2\right)}\left(k\right)={\left(1-\rho_{N}c_{CM}^{\left(2\right)}(k)\right)}^{-1},$$

(22) $$S_{CM}^{\left(3\right)}\left(k_{1},k_{2}\right)=S_{CM}^{\left(2\right)}\left(k_{1}\right)S_{CM}^{\left(2\right)}\left(k_{2}\right)S_{CM}^{\left(2\right)}\left(\left|k_{1}{+k}_{2}\right|\right)\left\{1+\rho_{N}^{2}c_{CM}^{\left(3\right)}(k_{1},k_{2})\right\},$$

where $c_{CM}^{\left(2\right)}\left(k\right)=c_{CM}^{\left(2\right)}\left(k\right)$ and $c_{CM}^{\left(3\right)}\left(k_{1},k_{2}\right)=c_{CM}^{\left(3\right)}\left(k_{1},k_{2},cos(k_{1},k_{2})\right)$ are the Fourier transforms of the functions $c_{CM2}\left(R_{1},R_{2}\right)=c_{CM2}(R_{12})$ and $c_{CM3}\left(R_{1},R_{2},R_{3}\right)=c_{CM3}\left(R_{12},R_{13},R_{23}\right),$ respectively. Equation (19) is the pair-level Ornstein–Zernike equation that defines the pair direct correlation function $c_{CM2}\left(R_{12}\right),$ which is a short-ranged function that shows a quick decay to zero with increasing distances [46]. Equation (20) belongs to Baxter’s exact hierarchy, under uniform changes in density [38,45], for the direct correlation functions in the quantum $c_{CMn}$ extension [19]. Equations (21) and (22) are the analytically closed reformulations of the pair and triplet structure factors defined in Equations (14) and (15). The advantage of Equations (21) and (22) is clear: none of them explicitly contain the simulation-intractable δ(k) terms [46] that appear in the “raw” analytic formulations based on the Fourier transforms of the $g_{2}$ and $g_{3}$ functions.

Note that all the structural functions depend on the density and the temperature, although such dependence is only written when necessary [20,27,60,61]. Equations (19)–(22) are mathematically exact and yield the formal solutions to the problematic zero-wavevector situations: (a) at the pair level, via $c_{CM}^{\left(2\right)}\left(k=0\right)$ [78]; and (b) at the triplet level, via Equation (20b) plus the symmetry property $c_{CM}^{\left(3\right)}\left(k_{1},k_{2}\right)=c_{CM}^{\left(3\right)}\left(k_{1},-k_{1}-k_{2}\right)$ for the cases $k_{1}=-k_{2}$ [45].

However, once the function $h_{CM2}\left(R_{12}\right)$ in real space is known (via PI simulation), the question arises as to how to determine the pair and triplet structure factors using the direct correlation functions $c_{CM2}(R_{12})$ and $c_{CM3}\left(R_{12},R_{13},R_{23}\right).$ These latter functions are defined by integral equations, and a hierarchical structure is in the way. In this regard, there are theoretical methods derived in the classical domain that, being based on closures, provide answers to these key problems [14,40,45,46,125,126,127,128]. These methods are highly accurate and reliable at the level n=2, and they can also be successfully applied to quantum calculations (regardless of the structural class!) [14,78,85,96,97]. However, the same favorable situation is not generally found at the level n=3, and neither in the classical case [44,45,47] nor in the quantum case [19,33,35]. In any event, one has two complementary routes to compute the foregoing structure factors: (a) the computationally “exact”, though incomplete (and expensive) route, based on the PI simulation of Equations (16) and (17)—or better, based on their more affordable translations into the canonical ensemble—and (b) the theoretically exact route, though subjected to the closure uncertainties in the triplet case, and based on the OZ3 framework condensed in Equations (19)–(22).

An observation of practical value is in order here. The standard PI simulation of the structural functions in **r**-space using the canonical ensemble, i.e., $Z_{PI}^{D}\left(N,V,T;\Psi =0\right)$, also poses well-known theoretical drawbacks (e.g., inadequate asymptotic behaviors and finite sample size) [19,81]. To cope with this general problem [26,46,126,127], some corrections [127,128] allow one to improve the pair-level canonical results, thus yielding good approximations to the grand canonical pair structures [34,35,81,96,97]. These corrections are based on the use of the Ornstein–Zernike OZ2 framework and go both ways: **r**-space ⇆ **k**-space, that is, involving jointly the pair of structures $\left\{g_{CM2},S_{CM}^{\left(2\right)}\right\},$ thereby benefiting the whole centroid pair structural determination issue.

#### 3.1.4. The Centroid Usefulness

Although centroid structures are not directly accessible via experimental techniques, it is important to make some further comments on the global usefulness of the centroid quantities. In addition to the abovementioned $w_{F}$ effective potential approximations, one finds the following applications of the set ${\left\{R_{CM,j}\right\}}_{j=1,2,\ldots ,N,\ldots }$:

(i) Keeping track of the closed paths (or necklaces) associated with the atoms *j* (or the one-site particles) throughout general PI simulations. Without any loss of generality, the closed paths can be expressed as $r_{j}\left(t\right)=R_{CM,j}+\xi_{j}\left(t\right),$ where use of the auxiliary general path-vector $\xi_{j}\left(t\right)$ is made (t runs over the bead labels 1,2,3,…,P). Thus, the displacements of the particles $∆r_{j}\left(t\right)$ can be referred to those of their moving centroids $∆R_{CM,j}$ plus those of the closed paths ${∆\xi }_{j}\left(t\right)$ around the centroids. This also yields the useful PI image of the centroid-constrained paths for every particle *j* [6,81,82,85,90,91,92,93,94,95,96,97,129]. In this regard, note that, when using SCVJ, some care is to be exercised to distinguish between the physically significant centroid of a necklace, involving the odd-numbered beads, and the auxiliary global “centroid” that can be defined for the whole set of *P* beads [14].
(ii) Fixing, via OZ2-$c_{CM2}\left(R\right),$ the equations of state for fluids with thermal quantum diffraction behavior [19,78,81], since in the grand canonical ensemble one finds:
  (23a) $$S_{CM}^{\left(2\right)}\left(k=0\right)=\frac{\langle N^{2}\rangle -{\langle N\rangle }^{2}}{\langle N\rangle }{=\rho }_{N}k_{B}T\chi_{T},$$
  where $\chi_{T}=$ $-\frac{1}{V}{\left(\frac{\partial V}{\partial p}\right)}_{T}$ is the isothermal compressibility.

(iii) Formulating higher-order number fluctuations; for example [19]:
  (23b) $$S_{CM}^{\left(3\right)}\left(k_{1}=0,k_{2}=0\right)=\frac{\langle {\left(N-\langle N\rangle \right)}^{3}\rangle }{\langle N\rangle }=\frac{\langle N^{3}\rangle }{\langle N\rangle }-3\langle N^{2}\rangle +2{\langle N\rangle }^{2},$$
  where the exact centroid relationships with OZ2 and OZ3 are to be noticed [32].

(iv) Characterizing global order in quantum samples through the use of centroid adapted quantities: Steinhardt et al.’s parameters [83], configurational pair structure factor information, etc. [6,34,82,97].
(v) Designing approaches to deal with quantum dynamics (e.g., centroid molecular dynamics [90,91,92,112] and other approximations [80,94]).
(vi) Dealing with coarse-graining approaches in quantum statistical mechanics [95].
(vii) Studying the number fluctuations under zero-spin BE conditions [19,78,79]. As a result of the action of an external field of constant strength $\Psi_{F},$ given the algebraic group character of the N! permutations, one can derive a partition function closely similar to Equation (11) involving the conventional PI centroids given by Equation (10):
  (24) $$\Xi_{PI}^{BE}\left(\Psi_{F}\right)=\sum_{N\geq 0}\frac{exp(\beta \mu N)}{N!}\int \left\{\prod_{j=1}^{N}dR_{j}\right\}\int \prod_{t=1}^{P}dr^{N,t}\times \left\{\prod_{j=1}^{N}\delta \left(R_{j}-R_{CM,j}\right)\right\}\times \;\underset{\Omega_{N}^{BE}}{\underbrace{{\sum_{P_{N}}C}_{N,P_{N}}exp\left\{-\beta W_{NP}\left(P_{N}\right)\right\}}}\times \exp\left(-\beta \sum_{j=1}^{N}\Psi_{F}(R_{j})\right),$$
  where the coefficients within the permutation sum are $C_{N,P_{N}}>0$, and the probability density function at zero field behaves as $\Omega_{N}^{BE}\geq 0$ [19,78]. Consequently, with the proviso that $\Omega_{N}^{BE}$ is used, number fluctuations can be counted as in Equation (23).

### 3.2. The Instantaneous Structures

Conceptually, the instantaneous ETn class is linked to the context of elastic scattering of radiation. The pair level ET2 can be understood by following the standard quantum treatments [21,27] of X-ray diffraction and inelastic neutron scattering. In X-ray scattering [22,27], the collisions of the X-ray photons with the electrons of the atoms define the nuclei positions, thereby yielding the actual pair quantum structures associated with this phenomenon: the radial correlation function $g_{Q2}\left(r\right),$ and the structure factor $S_{Q}^{\left(2\right)}\left(k\right)$ [9]. In neutron scattering [21,24,27], the neutron–nucleus interactions are described through Fermi’s pseudopotential (a δ—collision process); this time-dependent quantum phenomenon is characterized by the dynamic structure factor that, through its “elastic reduction” (frequency sum rule), also yields the same pair structures $g_{Q2}(r)$ and $S_{Q}^{\left(2\right)}\left(k\right)$ [27]. For both experimental techniques, the corresponding arguments belong to the general linear response theory [27], hence the statistical mechanics treatments give, in the end, the same response function from the fluid: $S_{Q}^{\left(2\right)}\left(k\right)$. Such a response is formulated in terms of $g_{Q2}(r)$ in the absence of the external field [79], the $S_{Q}^{\left(2\right)}\left(k\right)$ mathematical form being essentially the Fourier transform of $\left(g_{Q2}\left(r\right)-1\right)$ [21,22,27] (i.e., a “classical-like” formulation). Thus, one finds the instantaneous ensemble average recipes:

(25a) $$S_{Q}^{\left(2\right)}\left(k\right)=1+\rho_{N}\int dr\exp\left(ik\cdot r\right)\left(g_{Q2}\left(r\right)-1\right),$$

(25b) $${\rho_{N}^{2}g}_{Q2}\left(r_{12}\right)=\langle \sum_{j\neq l}\delta \left(r_{j}-q_{1}\right)\delta \left(r_{l}-q_{2}\right)\rangle ,$$

where $r=r_{12}=\left|q_{1}-q_{2}\right|.$ If the PI formalism is used, $g_{Q2}\left(r_{12}\right)$ directly translates into the following approximation [9,19,30,32]:

(26a) $$g_{Q2}\left(r_{12}\right)\approx g_{ET2}\left(r_{12}\right)=\frac{1}{X}\langle \sum_{t^{*}=1}^{X}\sum_{j\neq l}\delta \left(r_{j}^{t^{*}}-q_{1}\right)\delta \left(r_{l}^{t^{*}}-q_{2}\right)\rangle ,$$

The $t^{*}$ notation for beads is retained, and the associated structure factor reads as follows:

(26b) $$S_{Q2}^{\left(2\right)}\left(k\right)\approx S_{ET}^{\left(2\right)}\left(k\right)=1+\rho_{N}\int dr\exp\left(ik\cdot r\right)\left(g_{ET2}\left(r\right)-1\right),$$

where the grand canonical partition function $\Xi_{PI}^{D}$ at zero field is involved [19] as follows.

(27) $$\Xi_{PI}^{D}\left(\mu ,V,T;\Psi =0\right)=\sum_{N\geq 0}{exp\left(\beta \mu N\right)\;Z}_{PI}^{D}\left(N,V,T;\Psi =0\right).$$

(The forms of Equations (25) and (26) can be applied to both the grand canonical and the canonical ensembles [27], provided that the proper partition function is employed).

Although the basic PI canonical partition function $Z_{PI}^{D}$, Equation (5), already defines the beads that are going to be significant in the structure calculations, centroid-like functional calculus manipulations are inapplicable to the instantaneous class, this fact forces the Ψ=0 condition in Equation (27). If one wished to apply functional derivatives, setting Ψ≠0, to obtain the generalized structure functions $\Gamma_{ETn}$ (n=1,2,3,…), a fundamental difficulty would arise: the collision phenomena localize the actual quantum particles, which is not compatible with the structure of $Z_{PI}^{D}$ [19,79]. Within the current $\left\{r_{j}\right\}$ framework of nucleus positions, one faces the “collapse” of the atom quantum thermal packets, and the functional derivatives turn out to be meaningless; in fact, if this sort of manipulations were “formally” carried out, one would obtain the total thermalized-continuous linear response structures TLRn [19,32]. The functions $g_{ETn},$ $\Gamma_{ETn},$ and $S_{ET}^{\left(n\right)}$ at levels n>2 can be defined (using Ψ=0) by PI-adapting their classical counterparts (e.g., at n=3, Equations (12)–(18); see Appendix A.2 for related details) [19]. The reasons behind this procedure are as follows: (a) it is a straightforward consequence of the averaging of the dynamical *n*-body number density functions/operators (∏δ(r−q)) that define the elemental $g_{Qn}$ structures in real space [28] (e.g., Equation (25b)); and (b) consistency with the transition to the classical limit at every *n*-level for $\Gamma_{ETn}(\Psi =0$) [19]. In this work, attention will be focused on the triplet-level instantaneous structure functions $g_{ET3}(r_{12},r_{13},r_{23})$ and $S_{ET}^{\left(3\right)}\left(k_{1},k_{2},\cos\left(k_{1},k_{2}\right)\right).$

As remarked earlier, grand canonical PI simulations for obtaining structures pose a hard problem because of the computational effort involved. In general, the ETn class computations scale with X (i.e., *P*/2 for SCVJ, or *P* for CBHSP), and the practical alternative is based on the canonical ensemble applications [9,19,33,34,96]. Therefore, depending on the propagator selected, the instantaneous triplet structure averages read as follows [19,30,32]:

- SCVJ (fourth-order propagator)

(28) $${\rho_{N}^{3}g}_{ET3}\left(r_{12},r_{13},r_{23}\right)={\frac{2}{P}\langle \sum_{odd-t}\sum_{j\neq l\neq m\neq j}\delta \left(r_{j}^{t}-q_{1}\right)\delta \left(r_{l}^{t}-q_{2}\right)\delta \left(r_{m}^{t}-q_{3}\right)\rangle }_{Z_{PI}^{D}},$$

(29) $$S_{ET}^{\left(3\right)}\left(k_{1},k_{2}\right)=\frac{2}{NP}{\langle \sum_{odd-t}\sum_{j=1}^{N}\sum_{l=1}^{N}\sum_{m=1}^{N}exp\left[i\left(k_{1}\cdot \left(r_{j}^{t}-r_{m}^{t}\right)+k_{2}\cdot \left(r_{l}^{t}-r_{m}^{t}\right)\right)\right]\rangle }_{Z_{PI}^{D}}.$$

- CBHSP (pair action)

(30) $${\rho_{N}^{3}g}_{ET3}\left(r_{12},r_{13},r_{23}\right)={\frac{1}{P}\langle \sum_{t=1}^{P}\sum_{j\neq l\neq m\neq j}\delta \left(r_{j}^{t}-q_{1}\right)\delta \left(r_{l}^{t}-q_{2}\right)\delta \left(r_{m}^{t}-q_{3}\right)\rangle }_{Z_{PI}^{D}},$$

(31) $$S_{ET}^{\left(3\right)}\left(k_{1},k_{2}\right)=\frac{1}{NP}{\langle \sum_{t=1}^{P}\sum_{j=1}^{N}\sum_{l=1}^{N}\sum_{m=1}^{N}exp\left[i\left(k_{1}\cdot \left(r_{j}^{t}-r_{m}^{t}\right)+k_{2}\cdot \left(r_{l}^{t}-r_{m}^{t}\right)\right)\right]\rangle }_{Z_{PI}^{D}.}$$

The classical-like OZn framework for the instantaneous structures is not exact but an approximation [19,32,78]. At the pair level, however, OZ2 and its corrections to the canonical ensemble results have proven to be highly accurate in the thermal quantum diffraction regime [81,85,96,97,117]. Therefore, applications of OZ3 to the instantaneous triplet structures are well-worth exploring in depth [19,33,35]. In this context, the related basic equations can be directly translated from the centroid class (see Appendix A.3), for example:

(32) $$S_{ET}^{\left(3\right)}\left(k_{1},k_{2}\right)\approx S_{ET}^{\left(2\right)}\left(k_{1}\right)S_{ET}^{\left(2\right)}\left(k_{2}\right)S_{ET}^{\left(2\right)}\left(\left|k_{1}{+k}_{2}\right|\right)\left\{1+\rho_{N}^{2}c_{ET}^{\left(3\right)}\left(k_{1},k_{2}\right)\right\}.$$

Recall that the use of closures is compulsory at every OZn structural level [37,38,39,40,44,45,46,47,125,126,127,128]).

## 4. Triplet Closures and Associated Features

Given the classical-like forms of $\left\{g_{CM3},S_{CM}^{\left(3\right)}\right\}$ and $\left\{g_{ET3},S_{ET}^{\left(3\right)}\right\}$, the closures may be applied indistinctly to both of them. For the calculations carried out in this work, the employed closures are (a) for triplets in real space, Jackson–Feenberg convolution (JF3) [17] that contains Kirkwood superposition (KS3) [25], and the intermediate average AV3 [34], and (b) for triplets in Fourier space, Jackson–Feenberg convolution [17,40,45], and the symmetrized version of Denton–Ashcroft approximation (DAS3) [47]. Obviously, the pair information needed arises from PIMC simulations and OZ2 treatments.

The mathematical expressions for the real-space closures are given below:

- JF3
  (33a) $$g_{JF3}\left(d_{12},d_{13},d_{23}\right)=g_{KS3}\left(d_{12},d_{13},d_{23}\right)-h_{2}\left(d_{12}\right)h_{2}\left(d_{13}\right)h_{2}\left(d_{23}\right)+\;\;\rho_{N}\int dq_{4}h_{2}\left(d_{14}\right)h_{2}\left(d_{24}\right)h_{2}\left(d_{34}\right),$$
  where the distances $d_{ab}$ stand for $\left|R_{a}-R_{b}\right|$ for centroids or $\left|q_{a}-q_{b}\right|$ for actual quantum particles (the atoms of helium-3, or the one-site hard spheres), and $g_{KS3}$ is Kirkwood superposition [25], which reads as follows:

- KS3

(33b) $$g_{KS3}\left(d_{12},d_{13},d_{23}\right)=g_{2}\left(d_{12}\right)g_{2}\left(d_{13}\right)g_{2}\left(d_{23}\right).$$

- AV3

(34) $$g_{AV3}\left(d_{12},d_{13},d_{23}\right)=\frac{1}{2}\left(g_{KS3}\left(d_{12},d_{13},d_{23}\right)+g_{JF3}\left(d_{12},d_{13},d_{23}\right)\right).$$

The Fourier space closures read as follows:

- JF3

(35) $$c_{JF3}\left(d_{12},d_{13},d_{23}\right)=0\to \;c_{JF}^{\left(3\right)}\left(k_{1},k_{2}\right)=0.$$

- DAS3

(36a) $$c_{DAS}^{\left(3\right)}\left(k_{1},k_{2}\right)=\frac{1}{3}\left[c_{DA}^{\left(3\right)}\left(k_{1},k_{2}\right)+c_{DA}^{\left(3\right)}\left(k_{1},\left|k_{1}+k_{2}\right|\right)+c_{DA}^{\left(3\right)}\left(k_{2},\left|k_{1}+k_{2}\right|\right)\right],$$

$$c_{DA}^{\left(3\right)}\left(k_{1},k_{2}\right)=c_{DA}^{\left(3\right)}\left(k_{1},k_{2}\right)=$$

(36b) $$\frac{1}{c^{{\left(1\right)}^{\prime }}}\left\{c^{\left(2\right)}{(k}_{1})c^{{\left(2\right)}^{\prime }}{(k}_{2})+c^{\left(2\right)}{(k}_{2})c^{{\left(2\right)}^{\prime }}{(k}_{1})\right\}-\frac{c^{{\left(1\right)}^{\prime \prime }}}{{\left(c^{{\left(1\right)}^{\prime }}\right)}^{2}}c^{\left(2\right)}{(k}_{1})c^{\left(2\right)}{(k}_{2}),$$

(36c) $$c^{\left(1\right)\prime }=c^{\left(2\right)}\left(k=0\right),\;\;\;\;c^{{\left(1\right)}^{\prime \prime }}={\left(\frac{\partial c_{2}\left(k=0\right)}{\partial \rho_{N}}\right)}_{T},\;\;{\;\;c}^{{\left(2\right)}^{\prime }}\left(k\neq 0\right)={\left(\frac{\partial c_{2}\left(k\right)}{\partial \rho_{N}}\right)}_{T}.\;\;$$

This closure satisfies Baxter’s exact behavior Equation (20b) only at $k_{1}=k_{2}=0$.

The use of other closures to deal with triplet quantum calculations can be seen in References [19,29,30,31,32,33,34,35]. In particular, when considering Fourier space applications, AV3 and the elaborate Barrat–Hansen–Pastore factorization ansatz (BHP3) [45] appear to need very detailed numerical treatments [19,45]; this clarifying task is left for future work.

The structure factors $S_{CM}^{\left(3\right)}$ and $S_{ET}^{\left(3\right)}$ given by Equations (22) and (32), respectively, need their corresponding pair and triplet direct correlation functions (see Appendix A.3), and some remarks are necessary here. First, the pair direct correlation functions $c_{CM2}(R)$ and $c_{ET2}(r)$ can be determined in a highly accurate way [81,96,97] with the use of the OZ2 numerical procedure put forward by Baxter, Dixon, and Hutchinson (BDH) [125,126]. As stated earlier, the BDH application to $c_{CM2}(R)$ comes from an exact OZ2 framework, whereas application to $c_{ET2}(r)$ is an approximation. Second, the final closure results for $S^{\left(3\right)}$ are, in general, subjected to inaccuracies owing to the hierarchical structure of the direct correlation functions [38], which is illustrated for triplets by Equation (20). Third, the formal use of direct correlation functions yields answers to questions that cannot be exactly solved using PI simulations: the behaviors at zero-momentum transfers k=0 and $(k_{1}=0,k_{2}=0),$ which are contained in the theoretical relationships [19,32,78]:

(37a) $$S_{CM}^{\left(2\right)}\left(k=0\right)=S_{ET}^{\left(2\right)}\left(k=0\right)=\rho_{N}k_{B}T\chi_{T}={\left(1-\rho_{N}c_{CM}^{\left(2\right)}\left(k=0\right)\right)}^{-1},$$

(37b) $$S_{CM}^{\left(3\right)}\left(k_{1}=0,k_{2}=0\right)=S_{ET}^{\left(3\right)}\left(k_{1}=0,k_{2}=0\right)=S_{CM}^{\left(2\right)}\left(k=0\right)\left(S_{CM}^{\left(2\right)}\left(k=0\right)+\rho_{N}{\left(\frac{\partial S_{CM}^{\left(2\right)}\left(k=0\right)}{\partial \rho_{N}}\right)}_{T}\right).$$

It is worthwhile to make some useful observations here. (a) Equation (37) can be extended to include the TLR case [19,32,78], with $S_{TLR}^{\left(2\right)}\left(k=0\right)$ in Equation (37a) and $S_{TLR}^{\left(3\right)}\left(k_{1}=0,k_{2}=0\right)$ in Equation (37b). (b) Given the identities in Equation (37a), the ET and TLR extensions of Equation (37b) in terms of their pair $S^{\left(2\right)}(k=0$) components are equally valid. (c) Highly accurate estimates of the exact values can be obtained from the OZ2 centroid quantities, as Equation (37) arises from the consideration of the exact role played by $c_{CM}^{\left(2\right)}\left(k=0\right).$ (d) Although the analogous extensions of the whole Equation (37b) to ET and TLR are exact, their practical applications via OZ2 schemes will produce approximations to the exact behavior of the $S^{\left(3\right)}(0,0)$ quantity. And (e) by applying the underlying **k**-space symmetry properties [45], one can also obtain from Equation (20b) the exact values of the triplet CM3 components (k,−k):

(37c) $$c_{CM}^{\left(3\right)}\left(k,-k\right)=c_{CM}^{\left(3\right)}\left(-k,k\right)=c_{CM}^{\left(3\right)}\left(k,0\right)=c_{CM}^{\left(3\right)}\left(0,k\right)={\left(\frac{\partial c_{CM}^{\left(2\right)}(k)}{\partial \rho_{N}}\right)}_{T},$$

where the presence of single zero-momentum transfers is to be noticed. The general type of symmetry expressed in Equation (37c) allows one to set sum rules for the $S_{CM}^{\left(3\right)}\left(k,0\right)$ and $S_{ET}^{\left(3\right)}\left(k,0\right)$ components [32] (exact for the centroid case but approximate for the instantaneous case).

## 5. Computational Details

### 5.1. State Points

For the real system composed of helium-3 atoms, the atom mass is $m_{3_{He}}=3.01603\;amu$ and the critical point is located at ${(T}_{C}=3.3157\;K$; $\rho_{N,C}=0.0082246\;{Å}^{-3})$ [130]. The following supercritical state points are studied as follows [131]: SP1$\left(T=4.21\;K;\;\rho_{N}=0.0228717687\;{Å}^{-3}\right),$ SP2$\left(T=4.21\;K;\;\rho_{N}=0.0272988971\;{Å}^{-3}\right),$ SP3$\left(T=8.99\;K;\;\rho_{N}=0.0228717687\;{Å}^{-3}\right),$ and SP4$\left(T=4.2\;K;\;\rho_{N}=0.02286713\;{Å}^{-3}\right).$ At state points SP1, SP2, and SP3, the pair and triplet centroid correlation structures in **r**-space are investigated. At state point SP4, attention is focused on the evaluation of the instantaneous and centroid triplet response functions in **k**-space. In this connection, to carry out closure calculations, advantage is taken of previous work along the isotherm T=4.2 K [96], where the pair direct correlation functions were obtained at four state points about SP4 defined by the density variations $\pm ∆\rho_{N}=\pm 0.002{\;Å}^{-3},\;\pm 0.004{\;Å}^{-3}.$

For the model system composed of quantum hard spheres, the characterization of the state points only needs two parameters, namely the reduced density $\rho_{N}^{*}=\rho_{N}\sigma^{3}$ and the reduced de Broglie wavelength $\lambda_{B}^{*}=\frac{\lambda_{B}}{\sigma },$ and this fact unifies the description of this special system [34,132,133,134]. The state points on the crystallization line studied are as follows [82]: QHS1$(\lambda_{B}^{*}=0.2$; $\rho_{N}^{*}=0.789)$, QHS2$(\lambda_{B}^{*}=0.4$; $\rho_{N}^{*}=0.672)$, and QHS3$(\lambda_{B}^{*}=0.6$; $\rho_{N}^{*}=0.589)$. For comparison purposes, the actual parameter values used in these calculations are $m_{HS}=28.0134\;amu$ and σ=3.5 Å. In this regard, the temperatures and the number densities are QHS1($T=22.2024\;K;\rho_{N}=0.018402332\;{Å}^{-3}),$ QHS2($T=5.5506\;K;\;\rho_{N}=0.015673469\;{Å}^{-3}),$ and QHS3($T=2.4669\;K;\;\rho_{N}=0.013737609\;{Å}^{-3}).$ These state points are ascribed to the quantum diffraction regime. For example, note that at QHS3 one finds a low value for the degeneracy parameter $\gamma ={\left(\lambda_{B}^{*}\right)}^{3}\rho_{N}^{*}\approx 0.13.$ If fluid helium is modeled with QHS, even at ${(\lambda }_{B}^{*}=1.9832{;\;\rho }_{N}^{*}=0.348)$ that is on the crystallization line of this model [82,97,134], one would deal with temperatures far from FD or BE exchange interactions: (a) T≈5.3 K in helium-3, or (b) T≈4 K in helium-4 [65,134]. To carry out closure calculations, structure information at the pair level is needed. Consequently, two additional QHS state points about each of the listed interactions above are studied; $\lambda_{B}^{*}$ is kept constant and density variations $\pm {\Delta \rho }_{N}^{*}=\pm 1.715\times 10^{-2}$ are taken, which for comparison with the density variations in the helium-3 calculations amount to $\pm ∆\rho_{N}=\pm 0.0004\;{Å}^{-3}$. In addition, for completeness, four extra state points about QHS1, using the (smaller) uniform reduced density spacing $4.2875\times 10^{-3},$ are studied to fix their pair-level structures.

### 5.2. PIMC Simulations

The PIMC simulations of both systems follow the same patterns explained elsewhere [29,34,35,81,96,97]. However, for the reader’s convenience, the following operating procedures are worth mentioning. The canonical ensemble is selected, using a central cubic box of side *L* and sample size $N_{S}\times P$ (see below for the different choices made). The necklace normal-mode algorithm is utilized [75,85,135]. Therefore, any necklace in the simulation sample can move in *P* different modes: one of them is the translation of the necklace as a whole, and the rest (breathing modes or “collective excitations” of the beads [135]) describe independent ways for the *P* beads to displace by keeping their global “center of mass” fixed. (Recall the role of the relative path-vector $\xi_{j}\left(t\right);$ such global center of mass coincides with the CBHSP true centroid, Equation (10b), while it does not with the SCVJ true centroid, Equation (10a) [14]). In a uniform sampling of modes, this yields a reduced ratio $\left(\frac{1}{P}\right)$ for the centroid displacements, which indicates that the sampling of centroid quantities is less effective and will need more PIMC moves to attain reduced error bars. When evaluating structures, this fact becomes more critical in **k**-space than in **r**-space, because of the special features of the **k**-space sampling (see below) [46]. In building the Markov chains, the acceptance ratio for each of the different normal modes is set to 50%. The propagators are SCVJ $\left(\alpha =\frac{1}{3}\right)$ for helium-3, involving the SAPT2 interatomic potential [100], and CBHSP for quantum hard spheres. Given a simulation sample size $N_{S}\times P,$ the run lengths are quantified with the use of kpass and Mpass units: 1 kpass = $10^{3}N_{S}\times P$ attempted bead moves, 1 Mpass = $10^{6}N_{S}\times P$ attempted bead moves. The selected *P* values are known to be optimal [35,81,82,96,97], and the computations are focused on equilateral and isosceles features of the targeted instantaneous and (true) centroid triplet $g_{3}$ and $S^{\left(3\right)}$ structures. The specific details are given in the following sections.

#### 5.2.1. Real r-Space

The PIMC simulations of helium-3 utilize the following $N_{S}\times P$ values for the calculations of $g_{CM2}$ and $g_{CM3}:$ 1372×66 at SP1, 1372 × 80 at SP2, 1372×22 at SP3 (also, 1024×66 at SP4 [96]). (Results for the instantaneous $g_{ET2}$ and $g_{ET3}$ can be found in References [19,35,96]). The sampling of the pair and triplet structures in **r**-space uses a basic distance spacing ∆=0.1 Å for defining the histogram-bins of the functions [29,46]. The run lengths for the pair structures are 2000 kpasses at SP1, 1800 kpasses at SP2, and 1300 kpasses at SP3. The run lengths for the equilateral and isosceles triplet structures are 3660 kpasses at SP1, 3560 kpasses at SP2, and 2560 kpasses at SP3. (The simulation sampling at SP3 is enhanced with respect to Reference [35]: from 500 to 1300 kpasses for $g_{ET2\;},$ and from 750 to 2560 kpasses for $g_{ET3\;}).$ To fix the error bars (one-standard deviation), the run lengths are distributed in superblocks that give partial subaverages, which in turn serve to estimate the corresponding standard deviations. The resulting error bars are small. For example, in the close vicinities of the main peaks (max) obtained in the simulations, one finds that (a) at the pair-level $g_{CM2}(R_{max}$) and $g_{ET2}\left(r_{max}\right)$, the error bars are lower than 0.6% of the corresponding mean heights, and (b) at the triplet-level $g_{CM3}(R_{max},R_{max},R_{max}$) and $g_{ET3}\left(r_{max},r_{max},r_{max}\right),$ the error bars remain below 1% of the corresponding mean heights. The pair radial functions $g_{CM2}(R)$ (also $g_{ET2}(r)$ at SP3) are further processed with the OZ2 methodology [81,96] for fixing the pair structure factors $S_{CM}^{\left(2\right)}(k)$ (and $S_{ET}^{\left(2\right)}(k)$ at SP3); BDH variational calculations [125,126] are carried out, and their accuracy is increased with grand canonical corrections by using five Baumketner–Hiwatari iterations (BHw) [128]).

For the PIMC simulations, on the isothermal fluid branches, of the auxiliary QHS state points, i.e., those about the targeted on the crystallization line, the fixing of the pair radial structures $g_{CM2}$ and $g_{ET2}$ uses $N_{S}\times P=$ 864×12, and a spacing ∆=0.1 Å as the width of the histogram-bins. The run lengths are circa 3700 kpasses, and the error bars are very small (e.g., below 1% for the main peak heights).

#### 5.2.2. Fourier k-Space

These PIMC simulations utilize the following sample sizes $N_{S}\times P$: (a) for helium-3, 128×66 (SP4) and (b) for QHS, 250×12 (QHS1, QHS2, and QHS3). Note that for QHS the number of actual particles $N_{S}=250$ is greater than that of helium-3. The studied QHS state points are on the fluid side of the melting–freezing transition of this system, and the spurious fluid–solid flipping that is known to happen for small $N_{S}$ values [19,46] is to be avoided. Gaseous helium-3 state point SP4 does not present this problem, and $N_{S}=128$ serves well the purposes of this investigation.

The PIMC sampling of the triplet structures in **k**-space, for both helium-3 and QHS, involves the scanning of wave vectors that commensurate with the simulation box, i.e., $k=\left(\frac{2\pi }{L}\right)\left(k_{x}{,k}_{y},k_{z}\right),$ where $k_{x}{,k}_{y},$ and $k_{z}$ are integers. For the equilateral components $S^{\left(3\right)}(k_{q},k_{q},\frac{\pi }{3})$, twelve sets ${\left\{\left(k_{q1},k_{q2}\right);\ldots \right\}}_{q=1,\ldots ,12}$ are scanned, where each set is composed of eight pairs of wave vectors; these wave vectors have the same modulus, $\left|k_{q1}\right|=\left|k_{q2}\right|=\cdots =k_{q},$ and every pair defines an angle ϕ=π/3. The wave vectors are such that $2\leq k_{x}^{2}+k_{y}^{2}+k_{z}^{2}\leq 200,$ and it is easy to understand how the equilateral sets are built. For example, taking $k_{x}^{2}+k_{y}^{2}+k_{z}^{2}=32$, a pair of vectors is [(0,4,−4),(4,4,0)], another is [(0,4,4),(−4,0,4)], and so on. The corresponding ranges of simulated wavenumbers $k_{q},$ given in ${Å}^{-1}$ for comparison purposes, turn out to be (a) for helium-3, $0.5≲k_{q}/{Å}^{-1}≲5$ (SP4), and (b) for QHS, $0.74≲k_{q}/{Å}^{-1}≲3.72$ (QHS1), $0.70≲k_{q}/{Å}^{-1}≲3.53$ (QHS2), and $0.67≲k_{q}/{Å}^{-1}≲3.38$ (QHS3). For the isosceles components, only helium-3 at SP4 is investigated. In this regard, the performed analysis covers the components $S_{CM}^{\left(3\right)}(k_{m},k_{m},\phi )$ and $S_{ET}^{\left(3\right)}\left(k_{m},k_{m},\phi \right)$ defined at wavenumber $k_{m}=2.123236\;{Å}^{-1}.$ (Such value $k_{m}$ is near the maxima of the pair structure factors $S_{CM}^{\left(2\right)}(k_{M})$ and $S_{ET}^{\left(2\right)}\left(k_{M}^{\prime }\right)$, where $k_{M}\approx k_{M}^{\prime }\approx 2.06\;{Å}^{-1}$ [96]). Ten angles ${\left\{\phi_{i}\right\}}_{i=1,\ldots ,10},$ defined by groups of pairs $\left\{\left(k_{1},k_{2}\right)\right\}$ of modulus $k_{m},$ are scanned in the interval 0≤ϕ<π. For completeness, an extra eleventh component defined by $k_{m}^{\prime }=2.093539\;{Å}^{-1}$ and $\phi_{11}=2.077976\approx 2\pi /3$ is also added. Each angle $\phi_{i}$ contains in its sampling group a number of independent pairs of wave vectors, which are in between 4 and 16 in this work. It is also easy to see how the groups that define the angles $\phi_{i}$ are formed. For example, taking $k_{x}^{2}+k_{y}^{2}+k_{z}^{2}=36$, isosceles geometries at different angles $\phi_{i}$ can be obtained by extracting pairs of vectors from [(6,0,0);(0,0,−6);(4,4,2);(−4,4,−2);…]. (Recall that Equation (20b) and its instantaneous analog serve to determine the values of the $S^{\left(3\right)}(0,0)$ components and, also, of the isosceles components at ϕ=π).

It is important to stress that the PIMC simulations of triplet structure factors are subjected to large fluctuations. Therefore, in the present applications to helium-3 and QHS, the gathering of statistics for the **k**-space triplet structures is not equally distributed among the different components, as some of them receive an increased scanning attention (e.g., the components of maximal amplitude arising from the simulations).

The helium-3 run lengths are as follows. (a) Centroid case: in between 30 Mpasses and 409 Mpasses for the equilateral components, and in between 53 Mpasses and 278 Mpasses for the isosceles components. (b) Instantaneous case: in between 30 Mpasses and 120 Mpasses for the equilateral components, and in between 30 Mpasses and 48 Mpasses for the isosceles components. Helium-3 statistics are gathered from the Markov chain every 5000 configurations. As for QHS, the run lengths for the equilateral components are as follows. (c) Centroid cases: in between 6 Mpasses and 148 Mpasses. (d) Instantaneous cases: in between 6 Mpasses and 123 Mpasses. QHS statistics are gathered from the Markov chains every 8000 configurations. The statistical errors (one-standard deviation), fixed from block-subaverages, are found to remain controlled. For example, for helium-3 at the most sensitive of the equilateral components analyzed, that is, the obtained maximal peaks of the centroid and instantaneous structure factors (located at $k_{qm}\approx 2\;{Å}^{-1}),$ the error bars turn out to be ≈0.5% and 1.8% of the mean amplitudes $S_{CM}^{\left(3\right)}(k_{qm},k_{qm},\phi =\frac{\pi }{3})$ and $S_{ET}^{\left(3\right)}\left(k_{qm},k_{qm},\phi =\frac{\pi }{3}\right),$ respectively. As regards QHS, the situation is reasonably good but not so satisfactory: the error bars found for the equilateral maximal peak amplitudes, in both the centroid and the instantaneous cases, are circa 3.4% at QHS1, 5.4% at QHS2, and 5.6% at QHS3. (Complete results can be seen in the next Section).

### 5.3. Closure Calculations

The closure calculations with JF3 and AV3 in **r**-space (KS3 is trivial), and JF3 and DAS3 in **k**- space, are carried out in ways that have been described in detail elsewhere [29,30,31,32,33,34,35]. The main facts of interest to this work are given below.

#### 5.3.1. Helium-3

For helium-3, the following remarks are applicable.

(i) The $g_{CM3}(R,S,S)$ correlations, as approximated by the JF3 and AV3 **r**-space closures, involve the convolution integral included in Equation (33a). Such a convolution can be expanded as a Legendre polynomial series [29,43,45]; truncation, keeping the first thirty-one terms/polynomials $P_{\nu }\left(x\right),$ is utilized (−1≤x≤1;0≤ν≤30). The pair radial function $g_{CM2}(R)$ at each of the state points (SP1, SP2, and SP3) is used as data input, and the basic distance spacings for tabulating the triplet $g_{3}$ correlations are taken equal to 0.1 Å.
(ii) The pair structure factors $S_{CM}^{\left(2\right)}(k)$ and $S_{ET}^{\left(2\right)}\left(k\right)$ related to the helium-3 calculations are needed. In relation to this, OZ2 treatments apply BDH+BHw (5 iterations) to the pair radial structures obtained with PIMC [96] (see Reference [35] for calculations of $S_{ET}^{\left(2\right)}\left(k\right)\;$ at SP1, SP2, and SP3). However, for the triplet structure factors at SP4, the closure JF3 only requires the pair structure factors at this state point; DAS3 closure calculations do need their corresponding OZ2 direct correlation functions at the neighboring four state points to fix the corresponding isothermal density derivatives. The basic information on SP4 at the pair level is taken from the helium-3 study reported in Reference [96]. With regard to all these questions, the following OZ2-related information is worth giving here.

At a given state point, it is known that the combined method BDH + BHw, applied to a pair of functions $\left\{g_{2},S^{\left(2\right)}\right\},$ produces generally a sequence of solutions ${\left\{g_{2}\left(d\right);c_{2}\left(d\right);S^{\left(2\right)}\left(k\right);R_{Zi}\right\}}_{i=1,2,\ldots ,}$ [81]. These solutions are characterized by the values of the trial distances $R_{Zi}$ (the so-called “zeros”) that make $c_{2}\left(d\geq R_{Zi}\right)\equiv 0$. Practical convergence of the obtained functions is observed as the $R_{Zi}$ increase, and methods to deal with this feature have been discussed [81,96,97]. To carry out the related calculations in this article, the CM2 and ET2 solutions associated with their respective longest $R_{Zi}$ are selected. In this connection, it is worthwhile to compare the results at the most sensitive component, k=0 (i.e., the isothermal compressibility $\chi_{T}),$ also including the experimental data [131]. These values turn out as follows:

(a) At SP1, $\chi_{T}\left({bar}^{-1}\right)$ is 0.005288 for CM2 $\left(R_{Z}=18.507\;Å\right)$, 0.005202 for ET2 $\left(R_{Z}=16.974\;Å\right)$, with 0.005715 being the experimental value.
(b) At SP2, $\chi_{T}\left({bar}^{-1}\right)$ is 0.002401 for CM2 $\left(R_{Z}=17.108\;Å\right)$, 0.002844 for ET2 $\left(R_{Z}=15.187\;Å\right)$, with 0.002383 being the experimental value.
(c) At SP3, $\chi_{T}\left({bar}^{-1}\right)$ is 0.005526 for CM2 $\left(R_{Z}=16.083\;Å\right)$, 0.005552 for ET2 $\left(R_{Z}=16.738\;Å\right)$, with 0.005003 being the experimental value.

The present structural improvement achieved at SP3 explains the difference from its ET2 value with respect to Reference [35], where $\chi_{T}\left({bar}^{-1}\right)=$ 0.005071 was reported; as seen, the order of magnitude of $\chi_{T}$ does not change and a better agreement between the theoretical CM2 and ET2 estimates is obtained (consider Equation (37a) and its instantaneous analog involving $c_{ET}^{\left(2\right)}\left(k=0\right)$). Also, for completeness, the main amplitudes of the centroid and instantaneous pair structure factors at SP4 are as follows [96]: $S_{CM}^{\left(2\right)}\left(k_{M}=2.054\right)=1.947$, and $S_{ET}^{\left(2\right)}\left(k_{M}=2.067\right)=1.438.$

(iii) The isothermal density derivatives at SP4 needed for the **k**-space DAS3 calculations (CM3 and ET3) can be calculated through Richardson extrapolation [136], using four selected $c^{\left(2\right)}\left(k;\rho_{N};R_{Z}\right)$ functions, one per each of the auxiliary state points about SP4 (T=4.2 K and the uniform density spacing ${∆}_{b}\rho_{N}=0.002{\;Å}^{-3}$) [96]. By taking advantage of such uniform spacing, this algorithm reads as follows:
  (38a) $${\left(\frac{\partial c^{\left(2\right)}(k;\rho_{N})}{\partial \rho_{N}}\right)}_{T}\approx \frac{4D_{1}\left({∆}_{b}\rho_{N}\right)-D_{1}\left(2{∆}_{b}\rho_{N}\right)}{3},$$
  where, for a given density spacing $s_{\rho },$ the simple Stirling numerical estimates $D_{1}$ are given by the following:
  (38b) $$D_{1}\left(s_{\rho }\right)=\frac{c^{\left(2\right)}\left(k;\rho_{N}+s_{\rho }\right)-c^{\left(2\right)}\left(k;\rho_{N}-s_{\rho }\right)}{2s_{\rho }}.$$

In these DAS3 calculations at SP4, the selection of the $c_{2}\;$(and its associated $c^{\left(2\right)}$ and $S^{\left(2\right)})$ is made by utilizing these quantities at the longest $R_{Zi}$ zeros obtained via BDH+BHw(5 iterations). Such $R_{Z}$ zeros fall into $14.3<\frac{R_{Z}}{Å}<17.3$ for the centroid functions, and into $10.2<\frac{R_{Z}}{Å}<14.1$ for the instantaneous functions. Also, it is interesting to note that the values of the (four) real-space pair direct correlation functions at the origin (d=0) are such that they adhere to the following statement.

About SP4: $-29.8≲c_{CM2}\left(R=d=0\right)≲-10.9,$ and $-9.2≲c_{ET2}\left(r=d=0\right)≲-5,$

which indicate the depths of the respective $c_{2}-$“bowls”. To be highlighted are the significantly deeper depth and the larger variation range in the centroid case.

(iv) The basic wavenumber spacings for tabulating the $S^{\left(3\right)}$ components calculated with the closures are taken equal to 0.1 ${Å}^{-1}.$ The evaluations of the double-zero momentum transfer quantities $S^{\left(3\right)}\left(0,0\right)$ are carried out through Equation (37b) for the centroid cases using $S_{CM}^{\left(2\right)}\left(k=0\right),$ and its analogous (approximate) relationship for the instantaneous cases using $S_{ET}^{\left(2\right)}\left(k=0\right).$ Richardson extrapolations akin to Equation (38a) are employed for the density derivatives of the pair structure factor k=0 values. Alternatively, these quantities can be evaluated using the direct method based on the $c^{\left(2\right)}\left(k=0;\rho_{N}\right)$ values, Equations (20b) and (38a), etc. These two different methods, (i.e., based on $S^{\left(2\right)}\left(k=0\right)$ or $c^{\left(2\right)}(k=0)),$ are theoretically equivalent, although their numerical estimates may show slight differences because of the underlying density dependence of the input functions.

#### 5.3.2. QHS

Analogously, for the QHS **k**-space applications, the following items are to be remarked upon.

(i) For DAS3 calculations, the auxiliary QHS pair structural quantities needed are obtained via application of OZ2 to the PIMC $\left(N_{S}\times P=864\times 12\right)$ pair radial structures fixed in this work; applications of BDH+BHw (5- iterations) to the centroid structures, and of BDH alone to the instantaneous structures, are made. As mentioned earlier, OZ2 for the instantaneous correlations is an approximation, and the related application of the simplified BHw procedure in the region of the fluid–solid change of phase leads to unclear numerical behaviors of the iterations [97]. That is why BHw iterations are not included in the instantaneous analysis at any QHS state point. Whether more advanced grand canonical corrections [127] might cope with this drawback remains to be investigated. (Recall that for the JF3 application at a given state point only its pair structure factor is needed).
(ii) The DAS3 isothermal density derivatives for the cases CM3 and ET3 are calculated through the two-point differentiation Equation (38b) (calculations at state points QHS1, QHS2, and QHS3). This involves the $c^{\left(2\right)}(k;\rho_{N};R_{Z})$ functions obtained via OZ2, i.e., those arising from the sequences ${\left\{g_{2}\left(d\right);c_{2}\left(d\right);S^{\left(2\right)}\left(k\right);R_{Zi}\right\}}_{i=1,2,\ldots },$ about each QHS state point investigated ($\lambda_{B}=$ constant; basic density spacing $=0.0004\;{Å}^{-3},$ or $1.715\times 10^{-2}$ in reduced units). For each state point on the crystallization line, one representative solution of the foregoing CM2 or the ET2 sequences, is selected from the respective regions of significant convergence. The pair structure factors so fixed at the state points QHS1, QHS2, and QHS3 are employed in the final calculation of their triplet structure factors.

For the sake of comparison, it is worthwhile to quote the following QHS data associated with these OZ2 applications. The solutions selected correspond to $R_{Z}$ values that fall into the intervals: CM3 applications, $14<\frac{R_{Z}}{Å}<15;$ ET3 applications, $8<\frac{R_{Z}}{Å}<12.5$. These intervals are global in that they contain the $R_{Z}$ of all the state points: QHS1 and its two neighbors, QHS2 and its two neighbors, etc. Also, the depths of the real-space $c_{2}(d=0)-$“bowls” of the centroid and instantaneous cases adhere to the following statements.

(a) About QHS1, $-60.6≲c_{CM2}\left(R=0\right)≲-49.7,$ and $-52.8≲c_{ET2}\left(r=0\right)≲-44.7.$
(b) About QHS2, $-58.7≲c_{CM2}\left(R=0\right)≲-47.3,$ and $-45.1≲c_{ET2}\left(r=0\right)≲-37.3.$
(c) About QHS3, $-58.7≲c_{CM2}\left(R=0\right)≲-46.6,$ and $-36.8≲c_{ET2}\left(r=0\right)≲-30.9.$

The depths of these QHS “bowls” are significantly deeper than those at helium-4 state point SP4 (see Reference [19] for a discussion of the apparent negative repercussions of this feature on some closure-scheme evaluations). The wavenumber spacings for tabulating the $S^{\left(3\right)}$ components calculated with the closures are taken equal to $∆k^{*}=0.35$ (or $∆k=0.1{Å}^{-1}).$ The evaluations of the double-zero momentum transfer quantities $S^{\left(3\right)}\left(0,0\right)$ are carried out via the density derivatives of $c^{\left(2\right)}\left(k=0;\rho_{N}\right),$ by employing Stirling density derivatives (Equation (38b)).

(iii) As a complementary test, extended DAS3 calculations are carried out at QHS1 using five densities. On the isothermal fluid branch $\lambda_{B}^{*}=0.2,$ the state points about QHS1 at densities $\rho_{N}^{*}=0.789\pm 4.2875\times 10^{-3},\;\rho_{N}^{*}=0.789\pm 8.575\times 10^{-3}$ are also studied at the pair level with PIMC (864×12) and OZ2. The procedures for fixing the necessary CM2 and ET2 properties are the same as those described earlier, and the impact of this extension on the DAS3 results is discussed in the next Section.

## 6. Results and Discussion

### 6.1. Helium-3 Triplet Results in Real r-Space

**Figure 1:**
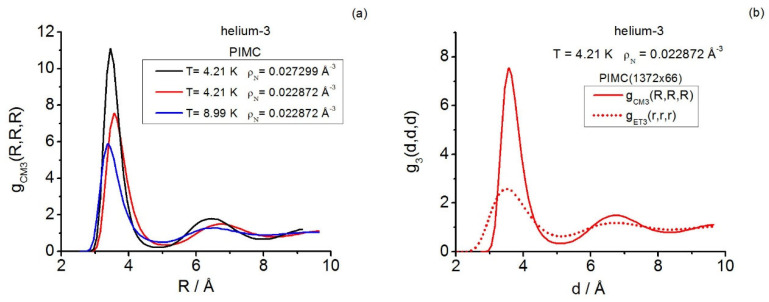
Path integral Monte Carlo (PIMC) equilateral correlations in supercritical helium-3. (**a**) Centroid correlations (CM3) at state points: SP1 $\left(T=4.21\;K;\rho_{N}=0.0228717687\;{Å}^{-3}\right),$ $N_{S}\times P=1372\times 66;$ SP2 $\left(T=4.21\;K;\rho_{N}=0.0272988971\;{Å}^{-3}\right),$ $N_{S}\times P=1372\times 80;$ SP3 $\left(T=8.99\;K;\rho_{N}=0.0228717687\;{Å}^{-3}\right),\;N_{S}\times P=1372\times 22;\;$(**b**) comparison between the centroid and instantaneous (ET3) correlations at SP1 (d stands for a general distance symbol: R for centroid distances and r for the instantaneous distances).

Figure 1 focuses on relevant equilateral PIMC results in **r**-space at state points SP1, SP2 and SP3. In Figure 1a one observes that more pronounced centroid features are obtained as the quantum effects become stronger (i.e., by increasing $\gamma =\lambda_{B}^{3}\rho_{N}),$ that is, SP2 > SP1 > SP3; at constant *T* (SP1 and SP2), the density marks the relative intensity of the diffraction effects, whilst at constant $\rho_{N}$ (SP1 and SP3) the inverse temperature does it. In this regard, see Figure 1b for a comparison between the centroid and instantaneous equilateral structures at SP1, where for instantaneous triplets (ET3) one notices a smaller distance of approach and a far smoother structure [19], as expected from delocalization arguments.

**Figure 2:**
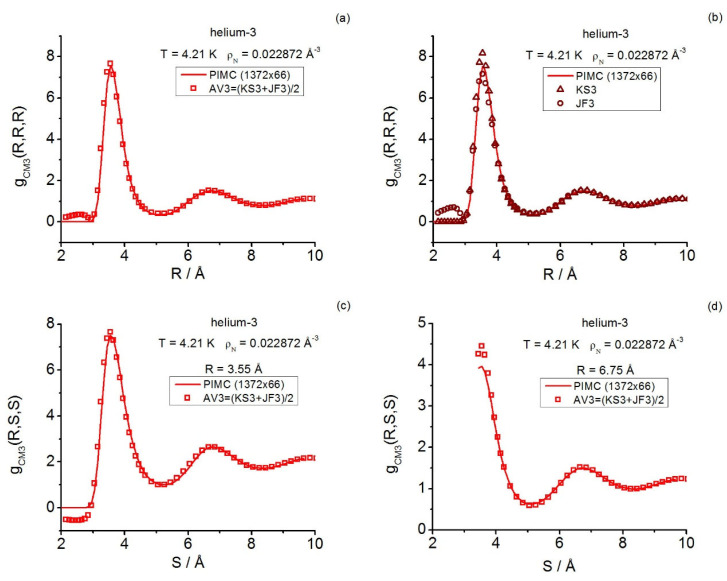
Path integral Monte Carlo (PIMC, $N_{S}\times P=1372\times 66$) and closure centroid correlation results in supercritical helium-3 at state point SP1$\left(T=4.21\;K;\rho_{N}=0.0228717687\;{Å}^{-3}\right)$. (**a**) Equilateral PIMC and intermediate closure AV3 Equation (34); (**b**) equilateral PIMC and closures KS3 (Kirkwood superposition) and JF3 (Jackson–Feenberg convolution), Equation (33); (**c**) isosceles PIMC and AV3 at slice R=3.55 Å; (**d**) isosceles PIMC and AV3 at slice R=6.75 Å.

**Figure 3:**
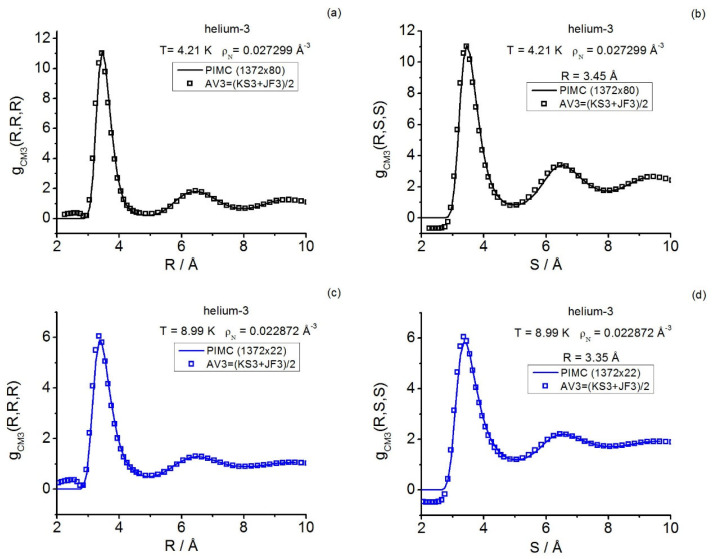
PIMC and AV3 closure centroid correlation results in supercritical helium-3. State point SP2 $\left(T=4.21\;K;\rho_{N}=0.0272988971\;{Å}^{-3}\right),$ $N_{S}\times P=1372\times 80:$ (**a**) equilateral; (**b**) isosceles at slice R=3.45 Å. State point SP3 $\left(T=8.99\;K;\rho_{N}=0.0228717687\;{Å}^{-3}\right)$, $N_{S}\times P=1372\times 22$: (**c**) equilateral; (**d**) isosceles at slice R=3.35 Å. Acronyms as in Figure 2.

Figure 2 displays, at state point SP1, typical features of the present centroid triplet correlations. Results obtained with PIMC, AV3, KS3, and JF3, are shown. Figure 2a,b focus on the equilateral $g_{CM3}(R,R,R)$ correlations: PIMC-AV3 comparison in Figure 2a, and PIMC-KS3-JF3 comparison in Figure 2b. As seen, AV3 yields excellent results for the range of significant-correlation distances (i.e., PIMC nonzero $g_{CM3}$ values), whereas neither KS3 nor JF3 does the work within the region of the main maximum (JF3 also fails within the range of short distances R≲3 Å; this failure influences the AV3 behavior) [34,35,60]). Figure 2c,d focus on some salient isosceles features of $g_{CM3}\left(R,S,S\right)$ by selecting the *R*-slices 3.55 Å and 6.75 Å, which are located in the vicinities of the equilateral main and secondary maxima, respectively. By discarding the negative influence of JF3 at short range, one observes that the overall performance of AV3 is excellent, although, as *R* increases, AV3 needs improvement in the short-range region of the available *S* distances (Figure 2d). For completeness, Figure 3 shows results for the PIMC centroid triplet correlations at state points SP2 and SP3, where trends analogous to those discussed above are observed. In addition, the updated results at SP3 obtained for the instantaneous $g_{ET3}(r,r,r)$ do not differ significantly on the scale of the graph from those previously reported in Reference [35], and they are not included in this work. More information on these triplet correlations is contained in several graphs within the Supplementary Material.

### 6.2. Helium-3 Triplet Results in Fourier k-Space

**Figure 4:**
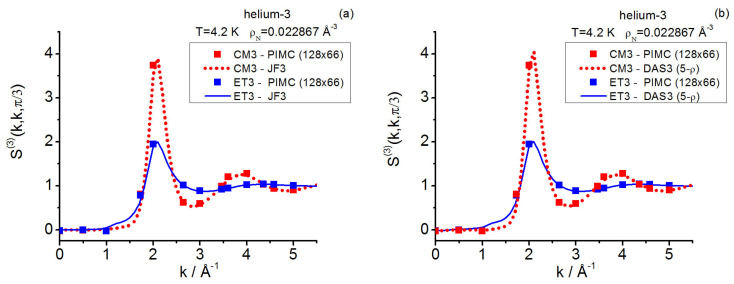
Fourier space results at supercritical helium-3 state point SP4 ($T=4.2\;K;\rho_{N}=0.02286713\;{Å}^{-3})$ obtained via path integral Monte Carlo (PIMC,$\;N_{S}\times P=128\times 66)$ and the closures: Jackson–Feenberg convolution JF3 [45] and symmetrized Denton–Ashcroft DAS3 [47]. Plots of the equilateral components of the triplet structure factors, centroid CM3 and instantaneous ET3, are given. Instantaneous PIMC and DAS3 results taken from the work in Reference [19], instantaneous JF3 results taken from the work in Reference [35]. (**a**) Comparison between PIMC and JF3. (**b**) Comparison between PIMC and DAS3. Closure direct correlation functions defined in Equation (35) for JF3 and Equation (36) for DAS3. Structure factors fixed with Equations (18) and (29) for PIMC, and Equations (22) and (32) for JF3 and DAS3. Pair-level data at the five state points studied in Reference [96] are used as input for DAS3 calculations.

**Figure 5:**
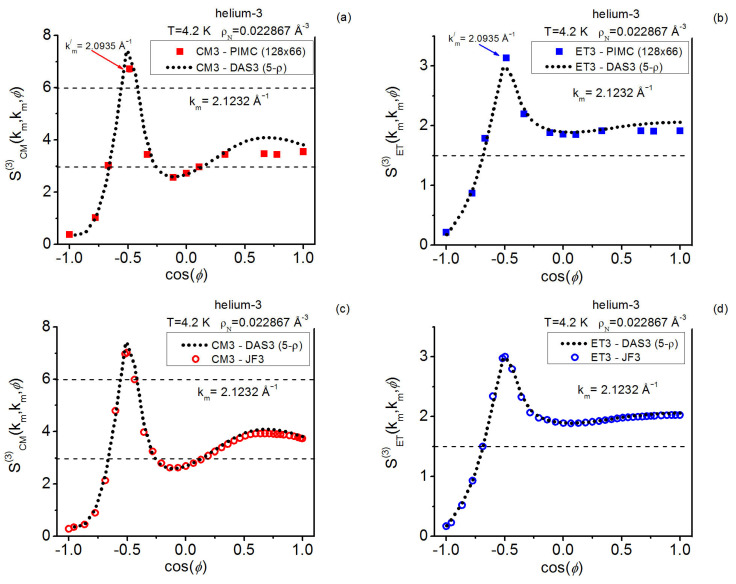
Fourier space results at supercritical helium-3 state point SP4 ($T=4.2\;K;\rho_{N}=0.02286713\;{Å}^{-3})$ obtained with path integral Monte Carlo (PIMC, $N_{S}\times P=128\times 66$), and the closures Jackson–Feenberg convolution JF3 [45] and symmetrized Denton–Ashcroft DAS3 [47]. Plots of the isosceles components of the triplet structure factors, centroid CM3, and instantaneous ET3 are given. (**a**) Centroid PIMC and DAS3 results. (**b**) Instantaneous PIMC and DAS3 results. (**c**) Centroid DAS3 and JF3 results. (**d**) Instantaneous DAS3 and JF3 results. Wavenumber $k_{m}=2.123236\;{Å}^{-1}$ in the vicinity of the main amplitude positions of both pair structure factors CM2 and ET2. (PIMC triplet main amplitudes in the vicinity of cosϕ=−0.5 fixed at $k_{m}^{\prime }=2.093539\;{Å}^{-1};$ explicitly marked in panels (**a**,**b**)). Pair-level data at the five state points studied in Reference [96] are used as input for DAS3 calculations. Acronyms as in Figure 4. The horizontal dashed lines are guides to the eye.

The computations carried out at state point SP4 are summarized in Table 1 and Table 2 and Figure 4 and Figure 5, where, as expected, the centroid features are more pronounced than the instantaneous ones. In more detail, from the intercomparison of the results obtained with PIMC, JF3, and DAS3, the following points can be remarked.

For the centroid and instantaneous equilateral components, $S_{CM}^{\left(3\right)}(k,k,\frac{\pi }{3})$ and $S_{ET}^{\left(3\right)}\left(k,k,\frac{\pi }{3}\right),$ the two closures produce results remarkably close to PIMC data, as shown in Figure 4 and Table 1. On the scale of the graphs, the corresponding amplitudes follow patterns only slightly different from each other, and the maxima and minima for $k≳2\;{Å}^{-1}$ are reasonably well located: see the comparisons PIMC-JF3 in Figure 4a and PIMC-DAS3 in Figure 4b (the resulting spacings ∆k used in the PIMC calculations preclude further analysis). A more detailed comparison can be seen in Table 1, which reveals some distinctive features. PIMC yields for the centroid and instantaneous cases negative values up to $k≲1\;{Å}^{-1}$, a trait that is obviously absent from JF3 (owing to its null $c_{3})$ but, most interestingly, not from DAS3. In particular, the sign of the double-zero momentum transfer is $S^{\left(3\right)}\left(0,0\right)=S_{CM}^{\left(3\right)}\left(0,0\right)<0$, as evaluated via Equation (37b) and the corresponding Richardson derivative of the type (38a). In this connection, it is interesting to compare the results obtained for $S_{CM}^{\left(3\right)}(0,0)$ and $S_{ET}^{\left(3\right)}\left(0,0\right)$ when using the two methods available: the based on the pair single zero-components $S_{CM}^{\left(2\right)}(k=0;\rho_{N})$ and $S_{ET}^{\left(2\right)}\left(k=0;\rho_{N}\right),$ and the based on the pair single zero-components $c_{CM}^{\left(2\right)}(k=0;\rho_{N})$ and $c_{ET}^{\left(2\right)}\left(k=0;\rho_{N}\right).$ Thus, one finds consistent results that indicate a negative value of the actual $S^{\left(3\right)}\left(0,0\right):$

(a) Method $S^{\left(2\right)}\left(k=0;\rho_{N}\right),$ Equation (37b) and its instantaneous analog,
  $S_{CM}^{\left(3\right)}\left(0,0\right)=-0.03211,$ and $S_{ET}^{\left(3\right)}\left(0,0\right)=-0.02153.$
(b) Method $c^{\left(2\right)}\left(k=0;\rho_{N}\right),$ Equation (20b) and its instantaneous analog,
  $S_{CM}^{\left(3\right)}\left(0,0\right)=-0.02755,$ and $S_{ET}^{\left(3\right)}\left(0,0\right)=-0.02111.$

In addition, the differences between JF3 and DAS3 are noticeable within the regions of low wavenumbers. DAS3 definitely takes negative values within: $k≲\;1.1\;{Å}^{-1}$ in the centroid case, and $k≲\;0.3\;{Å}^{-1}$ in the instantaneous case. The two closures JF3 and DAS3 stay very close to each other for wavenumbers: $k≳2.3\;{Å}^{-1}$ in the centroid case, and $k≳1.5\;{Å}^{-1}$ in the instantaneous case.

Figure 5 and Table 2 show the isosceles components $S_{CM}^{\left(3\right)}(k_{m},k_{m},\cos\phi )$ and $S_{ET}^{\left(3\right)}\left(k_{m},k_{m},\cos\phi \right)$, where $k_{m}\approx 2.12{\;Å}^{-1}$ is near the maximum-amplitude positions of the respective pair structure factors $k_{M}\approx 2.06\;{Å}^{-1}$ [96]. PIMC results are compared to those of DAS3 (panels (a) and (b)), and one observes that DAS3 and JF3 yield similar representations of the PIMC centroid and instantaneous behaviors (see also panels (c) and (d)); the closures appear to work better for smoother structures, as displayed in panels (b) and (d) that contain the instantaneous results. By separating the ϕ—angular dominion into two regions, $\Phi_{1}(-1\leq \cos\phi ≲0.4)$ and $\Phi_{2}(0.4≲\cos\phi \leq 1)$, the following features are to be noticed. Within $\Phi_{1}$, both closures capture reasonably well the shapes of the triplet CM3 and ET3 functions. (Recall that the PIMC main amplitudes located near cosϕ=−0.5 are fixed at wavenumber $k_{m}^{\prime }\approx 2.09\;{Å}^{-1},$ which is slightly different from $k_{M}$ and $k_{m}).$ Nevertheless, within $\Phi_{2}$, both closures depart from PIMC, which appears to show a leveled behavior (see also Table 2): the closures display larger amplitude values and a conspicuous undulating behavior in the centroid case. (For isosceles components at low wavenumbers, DAS3 yields negative values within certain angular ranges; not having PIMC-calculated components to be compared with, these results are not reported herein).

The current closure results for the instantaneous equilateral components are somewhat similar to those obtained with Barrat–Hansen–Pastore (BHP3) closure in Reference [35]. The instantaneous BHP3 isosceles components obtained in $\Phi_{2}$ also showed a leveled behavior that significantly stayed below most of the JF3 results. However, initial BHP3 calculations of the centroid triplet components at SP4 lead, as compared to PIMC, to not so fine results in general; there appear significant discrepancies that may be linked to the deeper bowl of the centroid pair direct correlation function quoted earlier (see Reference [19] for a related discussion). Therefore, no BHP3 results are reported in this article.

### 6.3. QHS Triplet Results in Fourier k-Space

**Figure 6:**
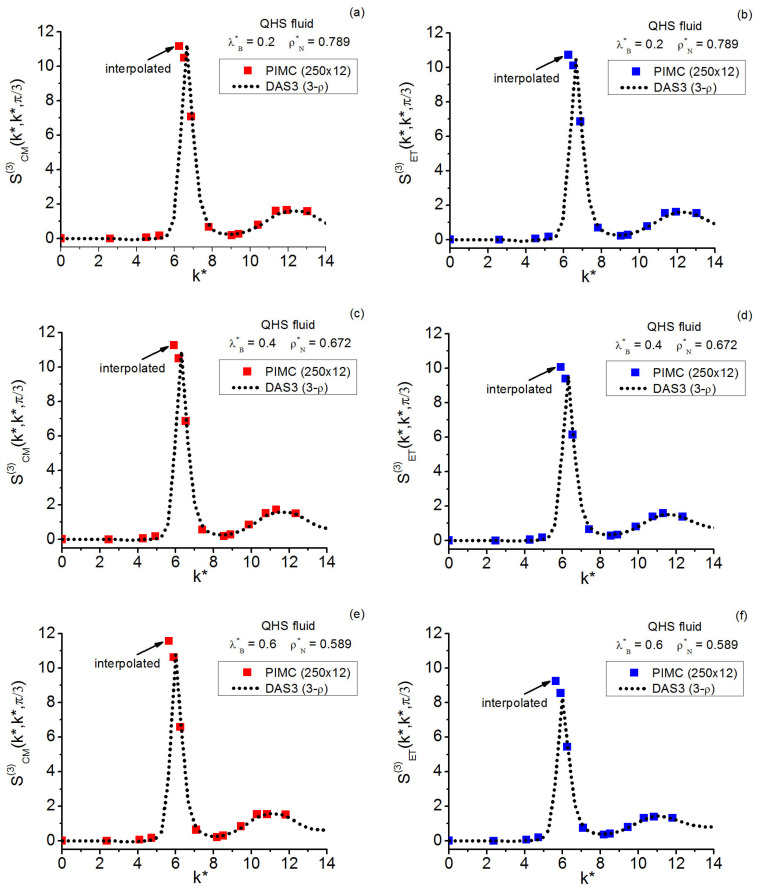
Fourier space quantum hard-sphere fluid (QHS) results. Plots of the equilateral components of the centroid and instantaneous triplet structure factors, $S_{CM}^{\left(3\right)}(k^{*},k^{*},\frac{\pi }{3})$ and $S_{ET}^{\left(3\right)}\left(k^{*},k^{*},\frac{\pi }{3}\right),$ are given. Results on the crystallization line [82] obtained with PIMC (250×12) and the symmetrized Denton–Ashcroft closure DAS3 [47]. State point QHS1 $(\lambda_{B}^{*}=0.2$; $\rho_{N}^{*}=0.789)$: (**a**) centroid CM3 components; (**b**) instantaneous ET3 components. State point QHS2 $(\lambda_{B}^{*}=0.4$; $\rho_{N}^{*}=0.672)$: (**c**) centroid CM3 components; (**d**) instantaneous ET3 components. State point QHS3 $(\lambda_{B}^{*}=0.6$; $\rho_{N}^{*}=0.589)$: (**e**) centroid CM3 components; (**f**) instantaneous ET3 components. PIMC absolute maxima obtained through quadratic interpolations. DAS3 calculations involve three state points along each of the fluid branches corresponding to the isotherms $\lambda_{B}^{*}=0.2,0.4,0.6,$ which are studied herein at the pair level with PIMC (864×12) plus OZ2 treatments.

**Figure 7:**
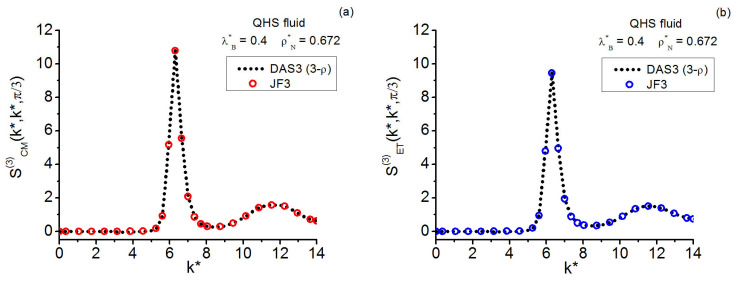
Closure results comparison at the quantum hard-sphere fluid state point QHS2 $(\lambda_{B}^{*}=0.4$; $\rho_{N}^{*}=0.672).$ Plots of the equilateral components of the triplet structure factors: (**a**) centroid $S_{CM}^{\left(3\right)}\left(k^{*},k^{*},\frac{\pi }{3}\right),$ and (**b**) instantaneous $S_{ET}^{\left(3\right)}\left(k^{*},k^{*},\frac{\pi }{3}\right).$ The closures Jackson–Feenberg convolution JF3 [45] and symmetrized Denton–Ashcroft DAS3 [47] are utilized.

Selected PIMC numerical results for the three state points, QHS1, QHS2, and QHS3, are given in Table 3. (More detailed numerical results are contained in the Supplementary Material). For visualization purposes, Figure 6 collects the computed equilateral components of the centroid and instantaneous triplet structure factors $S^{\left(3\right)}\left(k^{*},k^{*},\frac{\pi }{3}\right)$ computed with PIMC and DAS3; these results are plotted with the use of reduced units ${(k}^{*}=k\sigma ).$ Moreover, DAS3 and JF3 turn out to yield, on the scale of a graph, descriptions close to each other; Figure 7 displays a sample of this sort of general result illustrating the situation at QHS2, and the reader can also find more detailed information in the Supplementary Material (e.g., graphs at QHS1 and QHS3, DAS3 negative values within the region of low wavenumbers at QHS1, etc.).

Interestingly, at the revisited state point QHS3, the current PIMC mean maximal values are consistent with those previously reported in [19]. However, the extended sampling lengths involved in the current computations, which double those in [19], are not sufficient for reducing the corresponding error bars. Although the convergence of the Monte Carlo sampling is expected to behave with the inverse square root of the number of trials, the insensitiveness obtained is to be ascribed to the adverse role of the strong triplet fluctuations in **k**-space. This points to the expected magnitude of the task ahead when tackling this sort of triplet study on quantum crystallization lines. Also, recall that DAS3 calculations are based on the use of Equations (20b) and (38b), and their instantaneous analogs, which involve the groups of three state points studied with PIMC + OZ2 along each of the isotherms. For the three targeted fluid state points, one obtains values $S_{CM}^{\left(3\right)}\left(0,0\right)<0,$ which agrees with the independent similar behavior seen at the fluid helium-3 state point SP4 earlier; this behavior will be considered in more detail later on. The main features arising from all these $S^{\left(3\right)}\left(k^{*},k^{*},\frac{\pi }{3}\right)$ calculations are discussed in the points below:

(i) As compared to PIMC, the graphs indicate that DAS3 and JF3 appear to be slightly shifted towards larger wavenumbers (see also the Supplementary Material). The differences between DAS3 and JF3 are most noticeable within the low $k^{*}$-wavenumber region, since DAS3 gives negative values, whereas JF3 cannot yield that behavior. Despite the inaccuracies in the closure results, their current representations of the triplet structure factors turn out to be very valuable approximations to PIMC results. In the behavior of the equilateral components calculated with closures, JF3 takes over as $k^{*}$ increases (JF3 appears to yield a limit behavior).
(ii) From the graphs, one can observe at each state point that the triplet structure factor components show more pronounced features in the centroid $S_{CM}^{\left(3\right)}$ cases than in the instantaneous $S_{ET}^{\left(3\right)}$ cases. Furthermore, as the quantum effects increase (i.e., larger $\gamma =\lambda_{B}^{*3}\rho_{N}^{*},$ QHS1 < QHS2 < QHS3), one observes that the CM3-ET3 differences increase, and the structures are shifted towards lower wavenumbers. All of these traits are consistent with their analogs at the pair level when comparison of $S_{CM}^{\left(2\right)}(k)$ and $S_{ET}^{\left(2\right)}(k)$ is made [97].
(iii) As regards the centroid absolute maxima that can be derived from the equilateral PIMC results, if one uses a simple quadratic fitting of the mean amplitudes in the related regions, the obtained values hint (once again [33]) at the existence of a “constancy” in the maximal centroid equilateral amplitudes $S_{CM}^{\left(3\right)}\left(k_{M}^{*},k_{M}^{*},\frac{\pi }{3}\right)$. The related centroid estimates turn out to be (a) 11.153 (QHS1), (b) 11.259 (QHS2), and (c) 11.559 (QHS3). Roughly speaking, this would amount to having an interval ≈11.3±0.2 for the absolute maxima of the equilateral centroid components of the QHS model, which could be applicable within the present range of conditions investigated.
(iv) The foregoing centroid pilot result might be related to a hypothetical maximal $S_{CM}^{\left(3\right)}(k_{M},k_{M}^{\prime },\phi_{M})$-constancy for real systems that, because of the existence of triple point, show a liquid–solid coexistence line bounded from below in the $\left(T,\rho_{N}\right)—$plane (e.g., para-hydrogen, neon) [32,33,137,138]. However, to ascertain this question, one should carefully analyze the following issues:
  (a) The combined effects of a more exhaustive PI sampling, seeking to reduce the present error bars (≈3.5–5.5%), and a closer spacing $∆k^{*}$ for the fixing of the interpolating points; the sets of commensurate wave vectors should also include general triangular geometries.
  (b) The possible validity of such constancy hypothesis under stronger quantum effects; this implies to extend the study to a wider range of QHS fluid–solid coexistence conditions [82,134].
  (c) The situations presented by systems that, described by realistic interatomic potentials, present a noteworthy discordance between their associated crystallization lines, the modeled and the genuine [33,97].
(v) In relation to the instantaneous maximal equilateral components $S_{ET}^{\left(3\right)}\left(k_{M}^{*},k_{M}^{*},\frac{\pi }{3}\right),$ by quadratically fitting the PIMC data, as performed in the centroid case, one obtains the maximal amplitude estimates: 10.724 (QHS1), 10.060 (QHS2), and 9.238 (QHS3). Therefore, these maxima appear to decrease monotonically with increasing quantum effects. Regardless of the magnitude of the error bars and of the $∆k^{*}$ spacing available for defining these maximum regions, this behavior agrees with the general pattern of less-structured instantaneous functions arising when going from higher to lower temperatures and densities (QHS1 to QHS3) [33,34,97].
(vi) The existence of equilateral negative values for low wavenumbers is an observable fact for both classes of components, $S_{CM}^{\left(3\right)}$ and $S_{ET}^{\left(3\right)}.$ From the current calculations, PIMC results show just a glimpse of this behavior for $k^{*}≲2.6$ (Table 3), whilst DAS3 gives such a behavior within larger ranges, which go from $k^{*}≲4.9$ at QHS1-CM3 to $k^{*}≲3.5$ at QHS3-ET3 ${(∆k}^{*}=0.35).$ Furthermore, the double-zero momentum transfer components turn out to be negative, with the exception of the estimate $S_{ET}^{\left(3\right)}\left(0,0\right)=+0.00005$ at QHS1. (See the Supplementary Material for illustrative results at QHS1).
(vii) Given that density-differentiation algorithms that are more accurate than Equation (38b) can influence greatly the final estimates, particularly in situations involving zero-momentum transfers (i.e., values $k^{*}=0),$ this point deserves further consideration. Thus, it is worth noting that the additional DAS3 five-point closure calculations at QHS1, based on Equation (38a), do not show qualitative alterations in the magnitude of the foregoing CM3 and ET3 ranges of negative values resulting from Equation (38b). However, the use of Equation (38a) yields at QHS1: $S_{CM}^{\left(3\right)}\left(0,0\right)=-1.81\times 10^{-4}$ and $S_{ET}^{\left(3\right)}\left(0,0\right)=2.5\times 10^{-5},$ which are to be compared with the $k^{*}=0$ entries in Table 3 at QHS1: −0.0011 and 0.00005, respectively. (Recall that the calculation of the exact $S^{\left(3\right)}(0,0)$-component is obtained through the CM3 scheme). Finally, for completeness, the effect of the differentiation algorithm on DAS3 results becomes less important as $k^{*}$ increases, which can be observed within the maximal amplitude regions $k^{*}\approx k_{M}^{*}.$ For example, at QHS1 for $k_{M}^{*}=6.65$:
  (a) $S_{CM}^{\left(3\right)}\left(k_{M}^{*},k_{M}^{*},\frac{\pi }{3}\right),$ use of Stirling Equation (38b) yields 11.2462, whilst use of Richardson Equation (38a) yields 11.0824.
  (b) $S_{ET}^{\left(3\right)}\left(k_{M}^{*},k_{M}^{*},\frac{\pi }{3}\right),$ use of Stirling Equation (38b) yields 10.4487, whilst use of Richardson Equation (38a) yields 10.4435.

### 6.4. Further Comments and General Connections

The present investigation of quantum fluid triplet structures is just part of a first step in the topic. One real system and one model system, very different qualitatively, are analyzed in the diffraction regime (fluid helium-3 for γ≲3.2 and the quantum hard-sphere fluid for γ≲0.13). It is expected that, when the current computational-time constraints are not a serious problem, this sort of structural studies may benefit from the present developments.

In this regard, for monatomic fluid phases in the diffraction regime, key connections can be identified as follows: (a) The thermodynamic evaluations for fluids at nonzero temperatures based on the **r**-space structures (see References [20,48] for classical analogs). (b) For fluids on their crystallization lines, the finding of triplet order parameters that may extend the reach of the usual parameters employed at the pair level in the study of the melting–freezing transition (e.g., Hansen–Verlet rule [139], Steinhardt et al.’s $Q_{l}$ [83], angular parameters [140], Lindeman measures [141,142]), thus complementing the rigorous free-energy treatments [7,54,82,134,143,144,145]. (c) The exact centroid structural connections with theoretical frameworks for freezing [19,45,146,147]. And (d) the study of complex real systems at low temperatures: glass-forming liquids [62], metastable supercooled liquid states [143], or systems in which the interparticle repulsions dominate their behaviors.

As for general monatomic solid phases, the situation is far more involved [26,140,143] since the orientational features make the triplet structures be more complicated objects (e.g., 9-D functions in **r**-space). In addition, these studies should incorporate three-body interaction potentials when the effective pairwise approach cannot cope with very high-density conditions [9,148,149]. (When dealing with quantum hard spheres, the previous remark implies further intricacies to be faced [34]). However, in the study of monatomic crystalline/amorphous phases and/or melting–freezing phenomena [6,34,82,97,150], physically significant simulation results can be obtained at the pair-level by simplifying the treatment to the consideration of, not only the parameters $Q_{l}$ and Lindemann’s mentioned above, but also the pair radial structures and the pair configurational structure factors. (The latter are analyzed by taking configurational snapshots that serve to search for the structure factor maximal values). Then, it may be expected that the reduced 4-D triplet structures dealt with in this article may be useful for the characterization of solid phases in general, as shown in [34] for the quantum hard-sphere system.

Furthermore, given the recent developments in the treatment of monatomic Bose–Einstein fluids, via the path integral simulation methods PIMC [151] and PIMD [152], the related triplet structural research would be worth the endeavor [19]. Reasons for this expectation may be found in the role of triplet information in phonon–phonon interactions in superfluid helium-4 [17,18], and also in the exact connections of the centroid concept with BE statistics (see Reference [19] and Equations (23) and (24) herein, and References [88,89]). In relation to this, the behavior of the double-zero momentum transfer triplet component, $S^{\left(3\right)}\left(0,0\right),$ would be an interesting subject of study.

## 7. Conclusions

The present investigation on quantum fluid structures adds new triplet results to the knowledge of fluids composed of helium-3 atoms and of quantum hard spheres, for which initial information can be found in previous works by this author. Both systems are qualitatively very different, and they are studied under dissimilar thermal quantum diffraction conditions: supercritical for helium-3, and on the crystallization line for quantum hard spheres. Path integral Monte Carlo simulations and closure computations, at the pair and triplet levels of the centroid and instantaneous structures, are performed in the real and the Fourier spaces, and the following conclusions can be drawn.

In real space, the path integral triplet structures (equilateral and isosceles configurations) obtained for helium-3 show that the centroid structure features are more pronounced than their instantaneous counterparts, which is in agreement with the general expected behavior since centroids are known to mimic a fluid at a higher density than the actual one. The related whole path integral computational load depends critically on the simulation sample size $N_{S}\times P$ (e.g., in the canonical ensemble), which includes the number of necklaces (particles) $N_{S}$ and the number of beads P; even under strong quantum diffraction effects, this task is known to be perfectly affordable at the pair level, and by focusing on the abovementioned specific types of triplet configurations, it is also at the triplet level (run lengths ~3000 kpasses). Most interestingly, the application of the real-space intermediate closure AV3 to the determination of centroid and instantaneous triplet structures allows one, via comparison with exact path integral data, to confirm the great usefulness of this approximation: by analyzing the centroid correlations in helium-3, which bring about more extreme conditions than those previously studied for real systems, the AV3 performance is, once again, excellent and far less expensive than full path integral simulations.

AV3 is the straightforward average of two basic closures: Kirkwood superposition and Jackson–Feenberg convolution; both basic closures show clear deficiencies in the treatment of the triplet features in real space. Therefore, although AV3 partly inherits the unphysical short-range-distance failure of the Jackson–Feenberg convolution, the compensating effect of the abovementioned average makes AV3 capture most of the salient features of the path integral centroid correlations (equilateral and isosceles). Such a surprising triplet fact in real space was observed in recent works by this author (e.g., centroid and instantaneous triplets in the quantum hard-sphere fluid, and instantaneous triplets in supercritical helium-3). Within the significant-correlation ranges of distances (i.e., those showing nonzero path integral structure values), the AV3 applicability to fluids with quantum behavior appears to cover diffraction effects characterized by substantial values of the degeneracy parameter (γ≈3). However, note that AV3 worsens as the density increases along isotherms, and it does not capture relevant maximum-region behaviors in the isosceles configurations either. All in all, closures may give good and cost-effective “pictures” of complex interrelations among the actual atoms/particles in fluid phases. In this regard, the further centroid triplet results obtained in this work reinforce the idea that, in the study of quantum fluids via closures, the improvement of AV3 is well-worth exploring; this task should also incorporate general three-body configurations in the study. When trying to fix the abovementioned density effects that AV3 suffers from, the time-honored Abe’s developments may be a good guidance.

In the study reported in this work, the Fourier space applications to the fluid centroid and instantaneous triplet structures (equilateral and isosceles wave-vector geometries) illustrate the nature of these highly demanding calculations. In general, path integral Monte Carlo simulations present strong fluctuations in the structure factor component values along the Markov chain; this fact becomes critical in the vicinities of the main peak amplitudes investigated for both the centroid and the instantaneous structure factors. This imposes very long run lengths ($\sim 10^{2}–4\times 10^{2}$ Mpasses) to achieve reasonably reduced statistical errors in such regions. Such a hard computational situation is aggravated by some factors: (a) the number and sizes of the sets of commensurate wave vectors, which are needed to obtain significant descriptions of the components analyzed; and (b) the conditions under study, which put lower limits to the simulation sample size $N_{S}\times P$ to be utilized (i.e., in general, changes of phase influence $N_{S}$ and diffraction effects influence P). In this connection, selective sampling of the path integral structure factor components (e.g., equilateral main peak amplitudes) can be used for increasing the accuracy of the results in specific regions. In any event, currently, the whole quantum fluid triplet study in Fourier space via path integrals, even in the diffraction regime, with its diversity of classes and 4-D intricate numerical behavior, poses a formidable challenge in every respect. Accordingly, the use of closures in Fourier space is a useful way to obtain insights into the quantum triplet structure factor issues.

The path integral results clearly indicate that, for low and not large wavenumbers, the Fourier equilateral components take on (small) negative values in both the centroid and the instantaneous cases. This exact equilateral feature is observed at every fluid state point investigated in this work (helium-3 and quantum hard spheres). However, as regards the present closures, such a feature is only captured by the symmetrized Denton–Ashcroft closure and not in its full details (Jackson–Feenberg convolution cannot give this behavior). Thus, with respect to these below-zero regions, the centroid triplets are in general better represented than the instantaneous triplets by such Denton–Ashcroft closure, a feature to be ascribed to the classical-like structural nature of centroids (i.e., the exact theoretical applicability to centroids of Ornstein–Zernike schemes, in particular the pair OZ2). In addition, as compared to the data obtained via path integrals, both closures give reasonably good equilateral results beyond the below-zero regions, with Jackson–Feenberg convolution closure becoming dominant for large wavenumbers.

Continuing with the equilateral components, the path integral centroid calculations in Fourier space for the quantum hard-sphere fluid lead, via interpolation in the computed data tables, to equilateral main-peak amplitude estimates that suggest a “constancy” in these centroid quantities along the section of the crystallization line studied. This surmise needs more elaboration before stating its validity and range of applicability. Therefore, extended simulation work is needed to (a) reduce the current statistical errors, and (b) analyze more extreme quantum conditions. (As an aside, note that the analogous maxima of the instantaneous components cannot be candidates for “constancy”, because they show a definite decay pattern with increasing quantum effects). Moreover, other triplet centroid features in Fourier space (e.g., the maxima of salient isosceles components, as those near angles ϕ≈2π/3, etc.) might be subjected to this sort of “constancy” search. Even though the analysis of these possibilities entails costly simulations, this issue seems well-worth studying; its potential applicability to complex real systems, as a triplet way to provide more insights into quantum freezing phenomena, should be sufficient motivation. Furthermore, in the search for triplet-structure signatures of distinctive quantum fluid behaviors, the role of the centroid component $S_{CM}^{\left(3\right)}(0,0)$ should not be overlooked (recall its relationship with the third-order number fluctuations). The present results for the fluid phases analyzed give $S_{CM}^{\left(3\right)}\left(0,0\right)<0,$ a fact that indicates a negative skewness for all of the particle number (marginal) distributions involved in this work. Therefore, the ranges of values that this quantity may take under different thermodynamic conditions deserve further investigation; this is essentially a pair-level task, which could be undertaken by paying careful attention to the accuracy in the density derivatives involving just the centroid pair direct correlation functions.

For low wavenumbers, the isosceles components may be expected to take negative values (as suggested by closure calculations). However, the current path integral applications to helium-3 for the centroid and instantaneous structures, recorded at a wavenumber close to those of the maximum amplitudes of the respective pair structure factors, show that the exact path integral values stay above zero within the complete angular ϕ—range [0,π]. The symmetrized Denton–Ashcroft and Jackson–Feenberg convolution closures produce results remarkably close to the path integral treatment within the ϕ—subinterval [1.16,π], but they fail in its complement [0,1.16], where Jackson–Feenberg’s behavior shows a wrong trend that propagates into Denton–Ashcroft’s. The foregoing facts apply to both classes of isosceles results, centroid and instantaneous, although the discrepancies from the path integral reference values are especially pronounced in the centroid case (i.e., the closure significant undulations against the path integral leveled behavior). These results suggest that the study with other more numerically involved triplet closures (e.g., Barrat–Hansen–Pastore) may be highly significant and also help to clarify how to improve closure approaches.

The quantum fluid triplet topic presents one with many challenges, both theoretical and computational. The coming and extended use of increasingly efficient computational methods and hardware resources (i.e., exascale and, hopefully, quantum computers) can thrust the topic of fluid *n*-body structures forward, especially in the quantum domain. In any event, the research on triplet closures in both the real and the Fourier spaces will remain a valuable complement to the thorough understanding of the quantum fluid structures. It is expected that not only Statistical Mechanics but also disciplines involved in materials design can benefit from all these developments.

## Figures and Tables

**Table 1:** Helium-3 results at state point SP4 for the equilateral centroid and instantaneous components $S_{CM}^{\left(3\right)}\left(k,k,\frac{\pi }{3}\right)$ and ${\;S}_{ET}^{\left(3\right)}\left(k,k,\frac{\pi }{3}\right).$ PIMC = path integral Monte Carlo $\left(N_{S}\times P=128\times 66\right)$, DAS3 = symmetrized Denton–Ashcroft [47], JF3 = Jackson–Feenberg convolution [45]. Numbers in parentheses within the PIMC columns stand for one-standard deviation in the last decimal(s) of the mean value shown (e.g., 3.732(17) = 3.732 ± 0.017). Sampling of components is not uniform. Instantaneous PIMC, DAS3, and JF3 results obtained in the works reported in References [19,35]. DAS3 calculations use five state points along the isotherm T=4.2 K [96].

Helium-3 SP4 ($T=4.2\;K;\rho_{N}=0.02286713\;{Å}^{-3})$							
EQUILATERAL COMPONENTS $S^{\left(3\right)}\left(k,k,\frac{\pi }{3}\right)$							
	PIMC			DAS3		JF3	
$k/{Å}^{-1}$	$S_{CM}^{\left(3\right)}$	$S_{ET}^{\left(3\right)}$	$k/{Å}^{-1}$	$S_{CM}^{\left(3\right)}$	$S_{ET}^{\left(3\right)}$	$S_{CM}^{\left(3\right)}$	$S_{ET}^{\left(3\right)}$
0 ^a^	−0.0321 ^a^	−0.0215 ^a^	0 ^a^	−0.0321 ^a^	−0.0215 ^a^	0.0005	0.0006
0.500452	−0.010(1)	−0.008((1)	0.5	−0.0017	0.0128	0.0004	0.0029
1.000903	−0.033(1)	−0.028(2)	1	−0.0042	0.0607	0.0088	0.0474
1.733615	0.805(27)	0.782(4)	1.7	0.3975	0.5851	0.4311	0.5966
2.001806	3.732(17)	1.938(34)	2	3.6795	1.9096	3.5315	1.8934
2.648141	0.621(7)	1.008(1)	2.6	0.7025	1.0541	0.6966	1.0541
3.002710	0.594(18)	0.881(3)	3	0.5768	0.8809	0.5748	0.8807
3.467231	0.988(25)	0.915(1)	3.4	0.9284	0.9041	0.9287	0.9040
3.608808	1.201(9)	0.946(1)	3.6	1.1299	0.9458	1.1288	0.9458
4.003613	1.274(3)	1.019(2)	4	1.2378	1.0177	1.2371	1.0177
4.362836	1.031(22)	1.035(1)	4.4	1.0207	1.0326	1.0208	1.0326
4.586715	0.933(13)	1.025(3)	4.6	0.9289	1.0247	0.9287	1.0247
5.004516	0.897(7)	1.006(1)	5	0.8949	1.0046	0.8949	1.0046

^a^ Results based on the sum rules Equation (37b) and its instantaneous analog and the derivative algorithm Equation (38a).

**Table 2:** Fourier space results for helium-3 at state point SP4. Numerical values of the centroid and instantaneous isosceles components $S_{CM}^{\left(3\right)}\left(k,k,\phi \right)$ and $S_{ET}^{\left(3\right)}\left(k,k,\phi \right).$ Path integral Monte Carlo values (PIMC, $N_{S}\times P=128\times 66$) computed at ${\;k=k}_{m}=2.123236\;{Å}^{-1}$ (also $k_{m}^{\prime }=2.093539\;{Å}^{-1}$ in the vicinity of $\phi \approx 120^{o}).$ Closure values for the symmetrized Denton–Ashcroft (DAS3) [47] and the Jackson–Feenberg convolution (JF3) [45] fixed at ${\;k=k}_{m}$ (also $k_{m}^{\prime }).$ Numbers in parentheses within the PIMC columns stand for one-standard deviation in the last decimal(s) of the mean value shown (e.g., 6.72(13) = 6.72±0.13). Sampling of components is not uniform. DAS3 calculations use five state points along the isotherm T=4.2 K [96].

Helium-3 SP4 ($T=4.2\;K;\rho_{N}=0.02286713\;{Å}^{-3})$								
ISOSCELES COMPONENTS $S^{\left(3\right)}\left(k_{m},k_{m},\phi \right)$								
	PIMC		DAS3			JF3		
ϕ(°)	$S_{CM}^{\left(3\right)}$	$S_{ET}^{\left(3\right)}$	ϕ(°)	$S_{CM}^{\left(3\right)}$	$S_{ET}^{\left(3\right)}$	ϕ(°)	$S_{CM}^{\left(3\right)}$	$S_{ET}^{\left(3\right)}$
0	3.54(9)	1.91(1)	0	3.810	2.059	0	3.726	2.025
38.9424	3.43(6)	1.91(1)	38.499	4.063	2.043	38.499	3.896	2.013
48.1897	3.46(6)	1.91(1)	47.198	4.094	2.027	47.198	3.920	1.999
70.5288	3.43(9)	1.91(5)	72.133	3.467	1.931	72.133	3.379	1.924
83.6206	2.96(5)	1.85(5)	82.504	2.968	1.894	82.504	2.927	1.889
90	2.72(9)	1.85(4)	89.897	2.683	1.895	89.897	2.684	1.890
96.3794	2.55(9)	1.88(5)	97.801	2.591	1.941	97.801	2.621	1.946
109.4712	3.43(8)	2.20(4)	111.018	4.300	2.370	111.018	3.974	2.328
119.0593 ^a^	6.72(13)	3.13(8)	119.804 ^b^	7.435		119.758 ^d^		3.004
			119.398 ^c^		3.000	120.249 ^e^	7.014	
131.8103	3.02(12)	1.79(4)	133.389	2.344	1.485	133.389	2.131	1.500
141.0576	1.02(4)	0.86(4)	140.766	1.023	0.930	140.766	0.894	0.929
180	0.3647 ^f^	0.2133 ^f^	180	0.294	0.169	180	0.284	0.169

^a^ Near the PIMC maximum-amplitude angles for the isosceles configurations at $k=k_{m}^{\prime }$. ^b,c,d,e^ DAS3 and JF3 angles for the isosceles components of maximum amplitude at ${\;k=k}_{m}$. ^f^ Fixed with structure factor sum rules [32] based on Equations (20b) and (37c), and their instantaneous analogs, at ${\;k=k}_{m}$.

**Table 3:** Fourier space quantum hard-sphere fluid (QHS) results at state points QHS1$(\lambda_{B}^{*}=0.2$; $\rho_{N}^{*}=0.789),$ QHS2$(\lambda_{B}^{*}=0.4$; $\rho_{N}^{*}=0.672)$, and QHS3$(\lambda_{B}^{*}=0.6$; $\rho_{N}^{*}=0.589).$ Representative path integral Monte Carlo results (PIMC, $N_{S}\times P=250\times 12)$ for the centroid and instantaneous equilateral components, $S_{CM}^{\left(3\right)}\left(k^{*},k^{*},\frac{\pi }{3}\right)$ and $S_{ET}^{\left(3\right)}\left(k^{*},k^{*},\frac{\pi }{3}\right).$ $k^{*}=k\sigma$ (σ is the hard-sphere diameter). Results at QHS3 updated in this work with respect to those reported in Reference [19]. Numbers in parentheses stand for one-standard deviation in the last decimal(s) of the mean values shown (e.g., 10.49(34) = 10.49 ± 0.34; 0.249(7) = 0.249 ± 0.007). Sampling of components is not uniform.

PIMC SIMULATION								
EQUILATERAL COMPONENTS $S^{\left(3\right)}\left(k^{*},k^{*},\frac{\pi }{3}\right)$								
QHS1$(\lambda_{B}^{*}=0.2$; $\rho_{N}^{*}=0.789)$			QHS2$(\lambda_{B}^{*}=0.4$; $\rho_{N}^{*}=0.672)$			QHS3$(\lambda_{B}^{*}=0.6$; $\rho_{N}^{*}=0.589)$		
$k^{*}$	$S_{CM}^{\left(3\right)}$	$S_{ET}^{\left(3\right)}$	$k^{*}$	$S_{CM}^{\left(3\right)}$	$S_{ET}^{\left(3\right)}$	$k^{*}$	$S_{CM}^{\left(3\right)}$	$S_{ET}^{\left(3\right)}$
0	−0.0011 ^a^	0.00005 ^a^	0	−0.0011 ^a^	−0.0008 ^a^	0	−0.0009 ^a^	−0.0004
2.6068	−0.006(1)	−0.006(1)	2.4710	−0.006(1)	−0.007(1)	2.3647	−0.006(1)	−0.006(1)
4.5151	0.052(12)	0.052(11)	4.2798	0.044(12)	0.044(11)	4.0959	0.052(15)	0.053(13)
5.2136	0.159(20)	0.162(11)	4.9419	0.161(10)	0.168(12)	4.7295	0.157(12)	0.189(10)
6.5169	10.49(34)	10.10(35)	6.1774	10.50(55)	9.38(52)	5.9118	10.62(59)	8.54(48)
6.8969	7.07(22)	6.86(25)	6.5376	6.86(14)	6.13(12)	6.2565	6.57(22)	5.42(18)
9.3989	0.249(7)	0.272(6)	8.9092	0.262(14)	0.330(11)	8.5262	0.291(19)	0.401(8)
11.3627	1.587(15)	1.538(13)	10.7707	1.503(74)	1.387(47)	10.3077	1.527(18)	1.313(6)

^a^ Values fixed with the sum rules: Equation (20b) for centroid applications, and its analog for instantaneous applications; use of Equation (38b) is made.

## Data Availability

The original contributions presented in the study are included in the article and the Supplementary Material, further inquiries can be directed to the corresponding author.

## Supplementary Materials

The following supporting information can be downloaded at https://www.mdpi.com/article/10.3390/e28020231/s1, File SupplMat_S2026_LMS.pdf, containing: (A) **r**-space graphs of isosceles centroid correlations in helium-3 state points SP1, SP2, and SP3 (Figures S1 and S2); (B) PIMC and closure (DAS3, JF3) **k**-space numerical results for the quantum hard-sphere fluid at state points QHS1, QHS2, and QHS3 (Table S1 is an extension of Table 3 in the main text, Table S2 contains detailed closure numerical results at QHS1, and Figure S3 contains plots of closure results at QHS1 and QHS3).

## Appendix A

### Appendix A.1. Quantum Operator Manipulations

To obtain general operative forms for the partition function Equation (3), one applies to the density matrix operator the identity [9]:

(A1) $$\exp\left(-\beta H^{\left(N\right)}\right)=\overset{X\;factors}{\overbrace{\;\exp\left(-\beta H^{\left(N\right)}/X\right)\exp\left(-\beta H^{\left(N\right)}/X\right)\ldots \exp\left(-\beta H^{\left(N\right)}/X\right)\;}}.$$

Next, one inserts in Equation (A1) (X−1) spectral resolutions of the identity in the coordinate representation, i.e., $\int dr^{N,t^{*}}|r^{N,t^{*}}\rangle \langle r^{N,t^{*}}|=1$, where $t^{*}=1,2,\ldots ,X,$ $|r^{N,t^{*}}\rangle =|r_{1}^{t^{*}},r_{2}^{t^{*}},\ldots ,r_{N}^{t^{*}}\rangle$ and $dr^{N,t^{*}}={dr}_{1}^{t^{*}}dr_{2}^{t^{*}},\ldots ,\;dr_{N}^{t^{*}}.$ Then, by denoting $r^{N,1}\equiv r^{N},$ Equation (3) reads as follows:

(A2) $$Z^{D}\left(N,V,T;\Psi \right)=\frac{1}{N!}\int dr^{N,1}dr^{N,2}\ldots dr^{N,X}\left\langle r^{N,1}|exp(-\beta H^{\left(N\right)}/X)|r^{N,2}\right\rangle \times \;\left\langle r^{N,2}|exp(-\beta H^{\left(N\right)}/X)|r^{N,3}\right\rangle \left\langle r^{N,3}|exp(-\beta H^{\left(N\right)}/X)|r^{N,4}\right\rangle \ldots \left\langle r^{N,X}|\exp\left(-\beta H^{\left(N\right)}/X\right)|r^{N,1}\right\rangle .$$

Now, Equation (A2), which is an exact expansion, needs explicit forms for the propagator $\left\langle r^{N,t^{*}}|\exp\left(-\frac{\beta H^{\left(N\right)}}{X}\right)|r^{N,t^{*}+1}\right\rangle$, where the cyclic property $t^{*}+1=X+1\equiv 1$ is to be applied. In addition, for every atom *j*, the latter property brings forth discretized closed trajectories: $r_{j}^{t^{*}=1}\to r_{j}^{t^{*}=2}\to \cdots r_{j}^{t^{*}=X}\to r_{j}^{t^{*}=1}$. In the limit X→∞, any $r_{j}(t^{*}$) trajectory transforms into a closed continuous path $r_{j}\left(\tau \right)$ in imaginary time [2,9]. Therefore, any $r_{j}(t^{*}$) trajectory takes place in real space and evolves in imaginary time according to the “time”-step βℏ/X (see further details below). The foregoing general propagator, defined in the coordinate representation, takes nonnegative values [9], a decisive fact that guarantees the existence of probability densities in the related PI formulations. Note that the selection of the size of the imaginary time-step will influence the propagator form. Also, given that $H^{\left(N\right)}=H_{0}^{\left(N\right)}+\Psi^{\left(N\right)}=T^{\left(N\right)}+V^{\left(N\right)}+\Psi^{\left(N\right)},$ one must bear in mind that the kinetic energy operator $T^{\left(N\right)}$ does not commute with the potential energy operators $V^{\left(N\right)}$ and $\Psi^{\left(N\right)};$ hence, $H_{0}^{\left(N\right)}$ does not commute with $\Psi^{\left(N\right)}$ either.

Given that the PI partition function in the associated configurational space is consistent with a proper statistical analysis, the set of PI elastic closed paths is equivalent to a statistical distribution of closed paths. Factors that affect such statistical distribution are (a) the symmetry among the *N* descriptions ${\left\{r_{j}(t^{*})\right\}}_{j=1,2,\ldots ,N}$ of the particles, and (b) the packing of the particles and/or other parameters relevant for the system description [4,5,6,7,8,9,12,13,14]. In relation to this, surface effects are disregarded, and the actual bulk density of a system is taken as the number density $\rho_{N}$ (e.g., for *N* particles in a fixed volume V, the density is $\rho_{N}=\frac{N}{V}$).

The formulation of structures based on developments of Equation (A2) is a delicate matter, and one deals first with the questions involving $H_{0}^{\left(N\right)}$ and $\Psi^{\left(N\right)}$. One can proceed by applying the straightforward operator relationship [14]:

(A3) $$\exp\left(-\frac{\beta H^{\left(N\right)}}{X}\right)=\exp\left(-\frac{\beta }{X}\left[H_{0}^{\left(N\right)}+\Psi^{\left(N\right)}\right]\right)=\exp\left(-\frac{\beta \Psi^{\left(N\right)}}{2X}\right)\exp\left(-\frac{\beta H_{0}^{\left(N\right)}}{X}\right)\exp\left(-\frac{\beta \Psi^{\left(N\right)}}{2X}\right)+O\left(X^{-3}\right),$$

This arises from the Baker–Campbell–Haussdorf formula for the exponential operator built as the sum of two non-commuting operators [104]. Given that potential energy operators are diagonal in the coordinate representation, i.e., $\Psi^{\left(N\right)}|r^{N,t^{*}+1}\rangle =|r^{N,t^{*}+1}\rangle \Psi^{\left(N\right)}\left(r^{N,t^{*}+1}\right)$, $Z_{PI}^{D}$ in the main text, Equation (4), arises in a straightforward manner.

### Appendix A.2. Functional Derivatives and Centroid Linear Response

The functional derivatives of the grand canonical partition function Equation (11) with respect to the constant strength field $\Psi_{F}$ that give the first three general structures for centroids can be cast as follows [19,32]:

(A4) $$\left(-k_{B}T\right)\frac{\delta ln\Xi_{PI}^{D}\left(\Psi_{F}\right)}{\delta \Psi_{F}\left(R_{1}\right)}=\rho_{N,CM}^{\left(1\right)}\left(R_{1};\Psi_{F}\right)=\Gamma_{CM1}\left(R_{1};\Psi_{F}\right),$$

(A5) $${\left(-k_{B}T\right)}^{2}\frac{\delta^{2}ln\Xi_{PI}^{D}\left(\Psi_{F}\right)}{\delta \Psi_{F}\left(R_{1}\right){\delta \Psi }_{F}\left(R_{2}\right)}=\left(-k_{B}T\right)\frac{\delta \rho_{N,CM}^{\left(1\right)}\left(R_{1};\Psi_{F}\right)}{\delta \Psi_{F}\left(R_{2}\right)}=\Gamma_{CM2}\left(R_{1},R_{2};\Psi_{F}\right),$$

(A6) $${\left(-k_{B}T\right)}^{3}\frac{\delta^{3}ln\Xi_{PI}^{D}\left(\Psi_{F}\right)}{\delta \Psi_{F}\left(R_{1}\right){\delta \Psi }_{F}\left(R_{2}\right){\delta \Psi }_{F}\left(R_{3}\right)}=\left(-k_{B}T\right)\frac{\delta \Gamma_{CM2}\left(R_{1},R_{2};\Psi_{F}\right)}{\delta \Psi_{F}\left(R_{3}\right)}=\Gamma_{CM3}\left(R_{1},R_{2},R_{3};\Psi_{F}\right).$$

The foregoing equations define the generalized quantities $\Gamma_{CMn}$ (n=1,2,3), which contain the in-the-field correlation functions $g_{CMn^{\prime }}\left(R_{1},\ldots ,R_{n^{\prime }};\Psi_{F}\right)$ up to the very same upper level *n* $\left(n^{\prime }\leq n\right).$ Application of Yvon’s linear response developments [27,77] implies to take $\Psi_{F}=0$ on the right-hand sides of Equations (A4)–(A6). Thus, the trivial result at n=1 can be cast as follows:

(A7) $$\rho_{N,CM}^{\left(1\right)}\left(R_{1};\Psi_{F}=0\right)=\rho_{N}=\frac{\langle N\rangle }{V},$$

where $\rho_{N}$ is the mean number density for the isolated-from-Ψ quantum fluid. At the levels n=2 and n=3, one obtains the generalized zero-field centroid structures that, with the use of the total correlation function $h_{CM2}\left(R\right)=g_{CM2}\left(R\right)-1,$ can be written as follows [19]:

(A8) $$\Gamma_{CM2}\left(R_{1},R_{2};\Psi_{F}=0\right)=\rho_{N}^{2}h_{CM2}\left(R_{12}\right)+\rho_{N}\delta (R_{1}-R_{2}),$$

(A9) $$\Gamma_{CM3}\left(R_{1},R_{2},R_{3};\Psi_{F}=0\right)=\;\rho_{N}^{3}\left[g_{CM3}\left(R_{12},R_{13},R_{23}\right)-h_{CM2}\left(R_{12}\right)-h_{CM2}\left(R_{13}\right)-h_{CM2}\left(R_{23}\right)-1\right]+\;\rho_{N}^{2}\left[h_{CM2}\left(R_{12}\right)\delta (R_{2}-R_{3})+h_{CM2}\left(R_{13}\right)\delta (R_{1}-R_{2})+h_{CM2}\left(R_{23}\right)\delta (R_{1}-R_{3})\right]+\;\rho_{N}\delta \left(R_{1}-R_{3}\right)\delta \left(R_{2}-R_{3}\right).$$

At higher orders (n≥3), the process can be iterated giving the generalized correlation functions $\Gamma_{CMn}$ that describe, from the centroid standpoint, the loss of homogeneity of the quantum fluid brought about by $\Psi_{F}$. In general, one finds the linear response relationships (n≥1):

(A10) $${\left(-k_{B}T\right)}^{n+1}\frac{\delta^{n+1}ln\Xi_{PI}^{D}\left(\Psi_{F}\right)}{\delta \Psi_{F}\left(R_{1}\right)\ldots {\delta \Psi }_{F}\left(R_{n}\right){\delta \Psi }_{F}\left(R_{n+1}\right)}=\left(-k_{B}T\right)\frac{\delta \Gamma_{CMn}\left(R_{1},\ldots ,R_{n};\Psi_{F}\right)}{\delta \Psi_{F}\left(R_{n+1}\right)}\approx \Gamma_{CM,n+1}\left(R_{1},\ldots ,R_{n+1};\Psi_{F}=0\right),$$

(A11) $$\delta \Gamma_{CM}^{\left(n\right)}\left(k_{1},k_{2},\ldots ,k_{n};\Psi_{F}\right)\approx -\beta \delta \Psi_{F}\left(k_{1}+k_{2}+\cdots +k_{n}\right)\;\Gamma_{CM}^{\left(n+1\right)}\left(k_{1},k_{2},\ldots ,k_{n};\Psi_{F}=0\right),$$

(A12) $$S_{CM}^{\left(n+1\right)}\left(k_{1},k_{2},\ldots ,k_{n};\Psi_{F}=0\right)=\frac{1}{\rho_{N}}\Gamma_{CM}^{\left(n+1\right)}\left(k_{1},k_{2},\ldots ,k_{n};\Psi_{F}=0\right).$$

Equation (A11) results from Fourier transforming the center-right-hand side of Equation (A10), which takes advantage of the translational invariance [28] of the fluid structural quantity $\Gamma_{CM,n+1}(\Psi_{F}=0$). As seen, in Fourier space, the variation $\delta \Gamma_{CM}^{\left(n\right)}$ is a linear functional of the field applied.

### Appendix A.3. OZ3 Equations for the Instantaneous Case

The approximate OZ3 basic equations to be used in the instantaneous case, which complement Equation (32) in the main text, are summarized below $\left(r_{jl}=\left|q_{j}-q_{l}\right|\right)$.

(A13) $$h_{ET2}\left(r_{12}\right)\approx c_{ET2}(r_{12})+\rho_{N}\int dq_{3}c_{ET2}\left(r_{13}\right)h_{ET2}\left(r_{23}\right),$$

(A14) $${\left(\frac{\partial c_{ET2}(r_{12};\rho_{N})}{\partial \rho_{N}}\right)}_{T}\approx \int dq_{3}c_{ET3}\left(q_{1},q_{2},q_{3};\rho_{N}\right),$$

(A15) $${\left(\frac{\partial c_{ET}^{\left(2\right)}(k_{1};\rho_{N})}{\partial \rho_{N}}\right)}_{T}\approx c_{ET}^{\left(3\right)}\left(k_{1},k_{2}=0;\rho_{N}\right),$$

(A16) $$S_{ET}^{\left(2\right)}\left(k\right)\approx {\left(1-\rho_{N}c_{ET}^{\left(2\right)}\left(k\right)\right)}^{-1}.$$
