# Supplementary material for: Further Computations of Quantum Fluid Triplet Structures at Equilibrium in the Diffraction Regime
Source: Entropy (Basel). 2026 Feb 16;28(2):231. doi: 10.3390/e28020231 (PMC12939068; doi:10.3390/e28020231)
Supplement: Supplementary file 1 [file entropy-28-00231-s001.zip › entropy-4079119-supplementary.pdf]

Further Computations of Quantum Fluid Triplet Structures at Equilibrium in the Diffraction Regime

Luis M. Sesé

Independent Researcher, Sucursal 45 Correos, Avda. Valladolid 39, Apartado de Correos 45007, 28008 Madrid, Spain; msese@ccia.uned.es

Supplementary Material

Figures: S1, S2, and S3.

Tables: S1 and S2.

(A) Supercritical helium-3

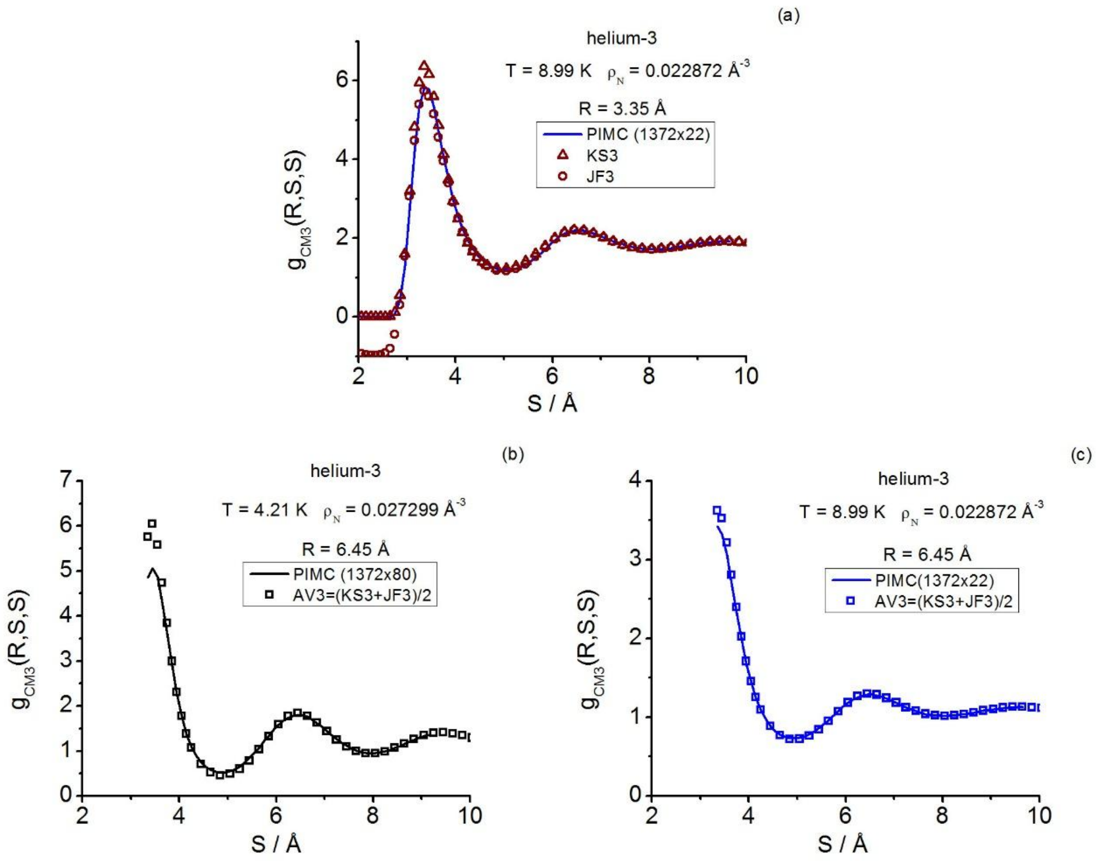

Figure S1. Helium-3 centroid isosceles correlations at different R slices obtained with PIMC and the KS3, JF3 and AV3 closures (KS3 = Kirkwood superposition, JF3 = Jackson-Feenberg, AV3 = intermediate). (a) State point SP3 (T = 8.99 K; ρ_N = 0.0228717687 Å^{-3}), slice R = 3.35 Å; (b) State point SP2 (T = 4.21 K; ρ_N = 0.0272988971 Å^{-3}), slice R = 6.45 Å; (c) State point SP3 (T = 8.99 K; ρ_N = 0.0228717687 Å^{-3}), slice R = 6.45 Å.

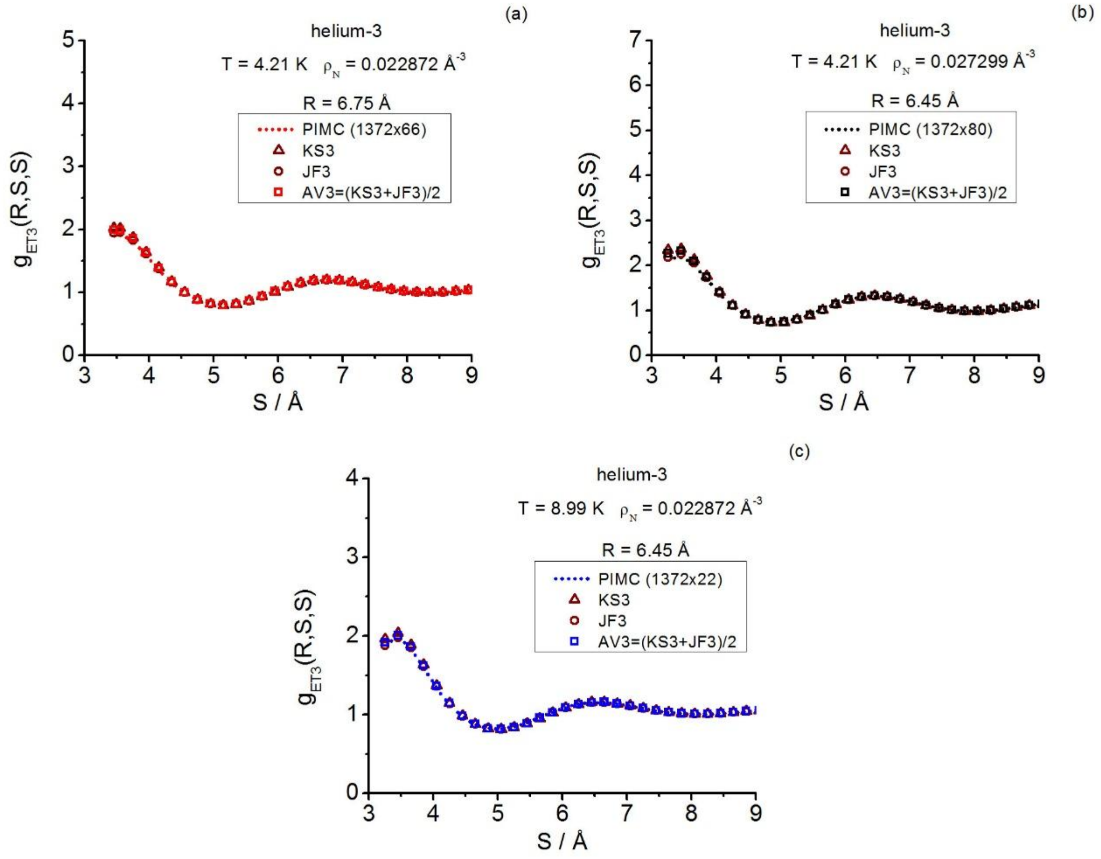

Figure S2. Helium-3 instantaneous isosceles correlations at different R slices obtained with PIMC and the KS3, JF3 and AV3 closures (KS3 = Kirkwood superposition, JF3 = Jackson-Feenberg, AV3 = intermediate). (a) State point SP1 (T = 4.21 K; ρ_N = 0.0228717687 Å^{-3}), slice R = 6.75 Å; (b) State point SP2 (T = 4.21 K; ρ_N = 0.0272988971 Å^{-3}), slice R = 6.45 Å; (c) State point SP3 (T = 8.99 K; ρ_N = 0.0228717687 Å^{-3}), slice R = 6.45 Å.

(B) Quantum hard-sphere fluid on the crystallization line

PIMC SIMULATION RESULTS FOR QHS

Table S1 (corresponds to an extended Table 3, but with wavenumbers in natural units).

Path integral Monte Carlo (PIMC, $N_S \times P = 250 \times 12$) results at the quantum hard-sphere fluid state points QHS1, QHS2, and QHS3, for the equilateral centroid $S_{CM}^{(3)}\left(k,k,\frac{\pi}{3}\right)$ and instantaneous $S_{ET}^{(3)}\left(k,k,\frac{\pi}{3}\right)$ triplet structure factors. Numbers in parentheses stand for one-standard deviation in the last mean value decimal(s) shown (e.g., 10.49(34) = 10.49 ±0.34; 1.485(69)=1.485±0.069). Hard-sphere diameter σ = 3.5 Å, mass m_{HS} = 28.0134 amu.

QHS1($\lambda_B^* = 0.2$; $\rho_N^* = 0.789$)			QHS2($\lambda_B^* = 0.4$; $\rho_N^* = 0.672$)			QHS3($\lambda_B^* = 0.6$; $\rho_N^* = 0.589$)		
$k/\text{Å}^{-1}$	$S_{CM}^{(3)}$	$S_{ET}^{(3)}$	$k/\text{Å}^{-1}$	$S_{CM}^{(3)}$	$S_{ET}^{(3)}$	$k/\text{Å}^{-1}$	$S_{CM}^{(3)}$	$S_{ET}^{(3)}$
0[a]	−0.0011	0.00005	0	−0.0011	−0.0008	0	−0.0009	−0.0004
0.74479	−0.006(1)	−0.006(1)	0.70599	−0.006(1)	−0.007(1)	0.67564	−0.006(1)	−0.006(1)
1.29002	0.052(12)	0.052(11)	1.22281	0.044(12)	0.044(11)	1.17024	0.052(15)	0.053(13)
1.48959	0.159(20)	0.162(11)	1.41198	0.161(10)	0.168(12)	1.35128	0.157(12)	0.189(10)
1.86198	10.49(34)	10.10(35)	1.76498	10.50(55)	9.38(52)	1.68910	10.62(59)	8.54(48)
1.97054	7.07(22)	6.86(25)	1.86788	6.86(14)	6.13(12)	1.78757	6.57(22)	5.42(18)
2.23438	0.663(79)	0.680(73)	2.11798	0.554(80)	0.632(62)	2.02692	0.627(37)	0.745(26)
2.58004	0.186(101)	0.213(92)	2.44563	0.173(69)	0.265(48)	2.34048	0.199(51)	0.349(30)
2.68539	0.249(7)	0.272(6)	2.54549	0.262(14)	0.330(11)	2.43605	0.291(19)	0.401(8)
2.97917	0.784(75)	0.778(67)	2.82397	0.841(65)	0.797(37)	2.70256	0.835(57)	0.783(33)
3.24648	1.587(15)	1.538(13)	3.07735	1.503(74)	1.387(47)	2.94505	1.527(18)	1.313(6)
3.41307	1.639(109)	1.603(89)	3.23526	1.720(13)	1.568(87)	3.09617	1.521(54)	1.381(29)
3.72397	1.576(34)	1.520(29)	3.52996	1.485(69)	1.367(37)	3.37820	1.511(18)	1.308(2)

[a] Values at $k = 0$ estimated via the two-point symmetric derivative (Stirling's) algorithm applied to each triplet-class of structure.

EXAMPLE OF CLOSURE NUMERICAL RESULTS FOR QHS

Table S2. Equilateral centroid and instantaneous triplet structure factors $S_{CM}^{(3)}\left(k,k,\frac{\pi}{3}\right)$ and $S_{ET}^{(3)}\left(k,k,\frac{\pi}{3}\right)$ fixed with the closures DAS3 and JF3 at QHS1($\lambda_B^* = 0.2$; $\rho_N^* = 0.789$). Hard-sphere diameter $\sigma = 3.5$ Å, mass $m_{HS} = 28.0134$ amu. DAS3 uses five points along the isotherm and Richardson extrapolation equation (38a); the uniform density spacing in reduced units is 4.2875×10^{-3}.

QHS1($\lambda_B^* = 0.2$; $\rho_N^* = 0.789$)				
	$S_{CM}^{(3)}\left(k,k,\frac{\pi}{3}\right)$		$S_{ET}^{(3)}\left(k,k,\frac{\pi}{3}\right)$	
$k/\text{Å}^{-1}$	DAS3	JF3	DAS3	JF3
0	−0.0002	7×10^{-6}	2×10^{-5}	4×10^{-6}
0.1	−0.0006	8×10^{-6}	-4×10^{-5}	4×10^{-6}
0.2	−0.0001	7×10^{-6}	−0.0003	5×10^{-6}
0.3	−0.0022	1×10^{-5}	−0.0006	6×10^{-6}
0.4	−0.0030	2×10^{-5}	−0.0012	1×10^{-5}
0.5	−0.0039	2×10^{-5}	−0.0021	2×10^{-5}
0.6	−0.0050	4×10^{-5}	−0.0033	4×10^{-5}
0.7	−0.0075	9×10^{-5}	−0.0055	9×10^{-5}
0.8	−0.0136	0.0002	−0.0101	0.0003
0.9	−0.0288	0.0008	−0.0224	0.0009
1	−0.0785	0.0042	−0.0642	0.0048
1.1	−0.1278	0.0138	−0.1103	0.0158
1.2	−0.0580	0.0125	−0.0488	0.0146
1.3	−0.0298	0.0141	−0.0210	0.0164
1.4	−0.0137	0.0242	−0.0029	0.0278

1.5	0.0217	0.0601	0.0381	0.0682
1.6	0.1781	0.2153	0.2098	0.2373
1.7	1.0758	1.0833	1.1226	1.1263
1.8	5.7152	5.6416	5.4760	5.4287
1.9	11.0824	11.1245	10.4435	10.4786
2	5.9105	5.9575	5.7962	5.8467
2.1	2.2963	2.3042	2.2716	2.2830
2.2	0.9761	0.9756	0.9734	0.9732
2.3	0.4898	0.4896	0.5034	0.5041
2.4	0.3086	0.3125	0.3240	0.3282
2.5	0.2474	0.2548	0.2631	0.2698
2.6	0.2487	0.2578	0.2673	0.2749
2.7	0.3018	0.3103	0.3249	0.3314
2.8	0.4098	0.4154	0.4360	0.4400
2.9	0.5699	0.5724	0.5946	0.5961
3	0.7720	0.7723	0.7868	0.7869
3.1	1.0103	1.0099	1.0019	1.0016
3.2	1.2627	1.2625	1.2249	1.2248
3.3	1.4641	1.4643	1.4243	1.4243
3.4	1.5662	1.5655	1.5623	1.5620
3.5	1.5962	1.5948	1.6148	1.6140
3.6	1.5916	1.5902	1.5787	1.5784
3.7	1.5258	1.5261	1.4653	1.4659
3.8	1.3591	1.3608	1.2938	1.2947
3.9	1.1232	1.1242	1.0942	1.0946
4	0.8951	0.8948	0.9050	0.9048
4.1	0.7236	0.7235	0.7561	0.7560
4.2	0.6185	0.6193	0.6596	0.6600
4.3	0.5723	0.5740	0.6151	0.6163
4.4	0.5794	0.5816	0.6204	0.6220
4.5	0.6403	0.6423	0.6753	0.6767
4.6	0.7581	0.7593	0.7802	0.7810
4.7	0.9290	0.9292	0.9289	0.9290
4.8	1.1273	1.1271	1.0982	1.0981
4.9	1.3018	1.3022	1.2471	1.2473
5	1.4000	1.4005	1.3339	1.3345

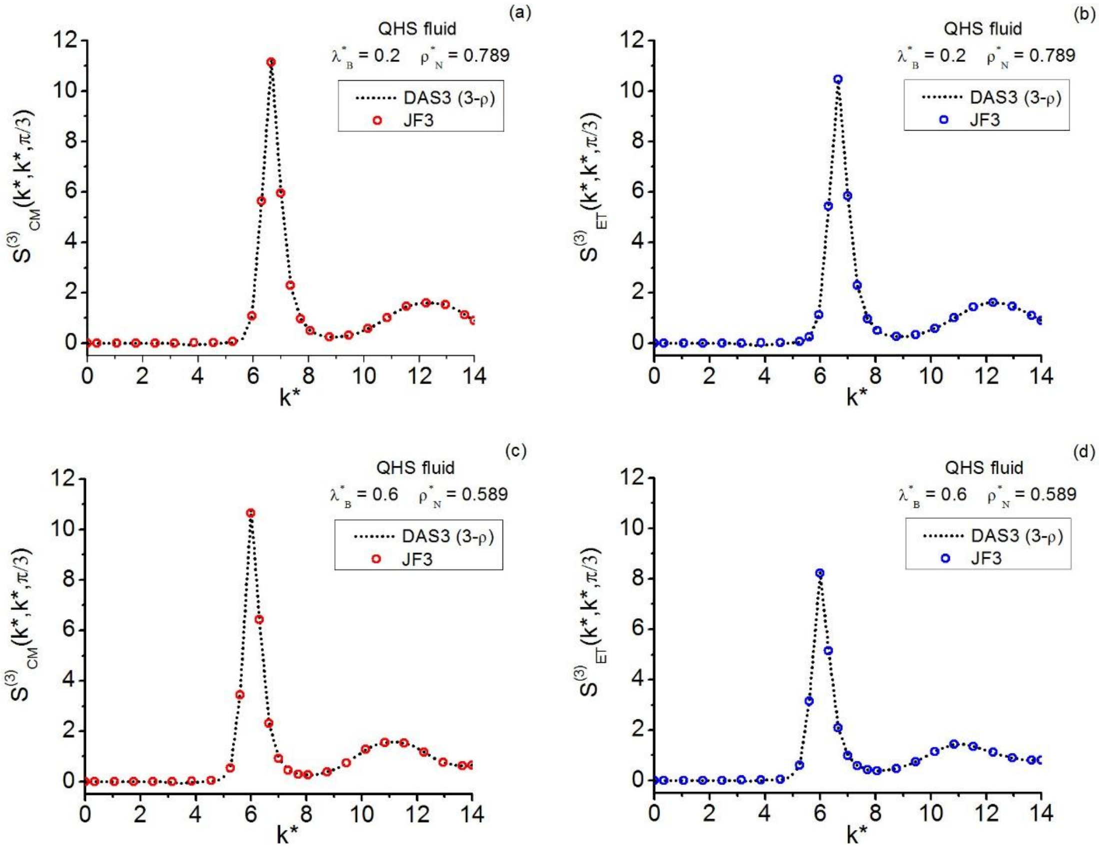

Figure S3. Quantum hard-sphere fluid results for the equilateral components of the centroid and instantaneous triplet structure factors obtained with the closures DAS3 (symmetrized Denton-Ashcroft) and JF3 (Jackson-Feenberg convolution approximation). State points on the crystallization line: QHS1 ($\lambda_B^* = 0.2$; $\rho_N^* = 0.789$) and QHS3 ($\lambda_B^* = 0.6$; $\rho_N^* = 0.589$) -the latter adapted from Reference [19] for the purposes of comparison-. $k^* = k\sigma$ is the reduced wavenumber and σ is the hard-sphere diameter ($\sigma = 3.5$ Å for the conversion of wavenumbers).
